# Alternative DNA secondary structure formation affects RNA polymerase II promoter-proximal pausing in human

**DOI:** 10.1186/s13059-018-1463-8

**Published:** 2018-07-12

**Authors:** Karol Szlachta, Ryan G. Thys, Naomi D. Atkin, Levi C. T. Pierce, Stefan Bekiranov, Yuh-Hwa Wang

**Affiliations:** 1 Department of Biochemistry and Molecular Genetics, University of Virginia, 1340 Jefferson Park Avenue, Charlottesville, VA 22908-0733 USA; 2 Relay Therapeutics, Inc., Cambridge, MA 02142 USA

**Keywords:** DNA secondary structure, RNA polymerase II promoter-proximal pausing

## Abstract

**Background:**

Alternative DNA secondary structures can arise from single-stranded DNA when duplex DNA is unwound during DNA processes such as transcription, resulting in the regulation or perturbation of these processes. We identify sites of high propensity to form stable DNA secondary structure across the human genome using Mfold and ViennaRNA programs with parameters for analyzing DNA.

**Results:**

The promoter-proximal regions of genes with paused transcription are significantly and energetically more favorable to form DNA secondary structure than non-paused genes or genes without RNA polymerase II (Pol II) binding. Using Pol II ChIP-seq, GRO-seq, NET-seq, and mNET-seq data, we arrive at a robust set of criteria for Pol II pausing, independent of annotation, and find that a highly stable secondary structure is likely to form about 10–50 nucleotides upstream of a Pol II pausing site. Structure probing data confirm the existence of DNA secondary structures enriched at the promoter-proximal regions of paused genes in human cells. Using an in vitro transcription assay, we demonstrate that Pol II pausing at HSPA1B, a human heat shock gene, is affected by manipulating DNA secondary structure upstream of the pausing site.

**Conclusions:**

Our results indicate alternative DNA secondary structure formation as a mechanism for how GC-rich sequences regulate RNA Pol II promoter-proximal pausing genome-wide.

**Electronic supplementary material:**

The online version of this article (10.1186/s13059-018-1463-8) contains supplementary material, which is available to authorized users.

## Background

While DNA is typically found in the B-DNA conformation, it has the ability to form a variety of non-B DNA secondary structures, including hairpins and quadruplexes. During DNA processes like replication and transcription, the duplex DNA is unwound, potentially allowing single-stranded DNA to form stable secondary structures, including stem-loop structures. Once formed, DNA secondary structures can play a role in many processes, including replication, transcription, and DNA repair [1]. The extent of DNA secondary structure formation at a genome-wide level is still not fully understood, but studies have demonstrated the importance of DNA secondary structure formation at a number of genomic loci. DNA hairpins formed during V(D)J recombination protect the ends of coding sequences prior to processing by Artemis:DNA-PKcs [2, 3]. DNA secondary structures can also influence DNA-modifying enzyme activity, either by protecting DNA [4, 5] or by promoting enzyme activity [6, 7]. A variety of DNA structures formed in gene promoter regions have been suggested to block gene expression for a number of genes [8–14]. Conversely, DNA secondary structure can promote transcription by altering transcription factor binding sites [15].

During transcription, RNA polymerase II (Pol II) binds and initiates transcription, but only travels within 100 nucleotides (nt) downstream of the transcription start site before stalling in a process known as promoter-proximal pausing [16–21]. While the full mechanisms leading to promoter-proximal pausing and the eventual release into productive elongation are still not fully understood, several protein factors, as well as the DNA and RNA sequences themselves, have been shown to contribute [8, 10, 22–28]. Several studies have postulated that CpG islands and local GC-rich sequences typically found in the promoter-proximal region of many genes might serve as energy barriers due to the stronger duplex DNA sequence formed by G–C base pairs [18, 29]. Alternatively, GC-rich sequences are likely to form stable DNA secondary structure, which could provide a mechanism for how high GC content in the promoter-proximal regions can influence Pol II pausing.

Formation of stable DNA secondary structures, such as quadruplex, can occur throughout the genome [30], and sequence motifs for quadruplexes are enriched in the non-template strand of the region flanking the transcription start site (TSS) among pausing genes [31]. However, genome-wide analysis of DNA secondary structure formation has been limited to algorithms able to identify specific sequence motifs that have the potential to form specific structures, such as the *quadparser* program for quadruplex structures [32], but do not provide stability measurements to rank the structure-forming sequences. Software platforms such as Mfold [33] and ViennaRNA [34] are able to calculate the Gibbs free energy (ΔG) of DNA secondary structures for given sequences, and therefore not only identify sites of potential DNA secondary structure formation, but also provide a measure of structural stability. Further, new methods to measure pausing of Pol II have been developed based on functional genomics data: Pol II ChIP-seq, Global Run on Sequencing (GRO-seq) [35], Native Elongating Transcript sequencing (NET-seq) [36], and mammalian-Native Elongating Transcript sequencing (mNET-seq) [37].

With these tools and datasets in hand, we analyzed the human genome for sites of highly stable DNA secondary structure using the Mfold and ViennaRNA programs with thermodynamic parameters for DNA analysis. We demonstrated that loci with high propensity to form stable secondary structures are highly correlated with loci displaying robust hallmarks of Pol II pausing. Using the combination of Pol II ChIP-seq, GRO-seq, NET-seq, and mNET-seq signals, we refined Pol II pausing sites at nearly single nucleotide resolution, and by overlaying an average free energy profile, we found highly stable secondary structures located ~ 10–50 nt upstream of the pausing sites. Further, analyzing data from two DNA secondary structure-probing experiments performed in human cells [38, 39], we revealed that DNA secondary structures are indeed enriched in the promoter-proximal regions of Pol II pausing sites, which provides strong validity to the application of the Mfold and ViennaRNA programs. Finally, we demonstrated that manipulation of stem-loop structures upstream of the Pol II pausing site in the human *HSPA1B* gene affects Pol II pausing in an in vitro transcription system with strong pausing associated with more stable DNA secondary structure. Our results demonstrate the potential for alternative DNA secondary structures to play a role in the regulation of gene expression by contributing to the pausing of Pol II during transcription elongation.

## Results

### DNA secondary structure formation is prevalent throughout the human genome and associates with RNA polymerase II binding at transcription start sites

**Fig. 1:**
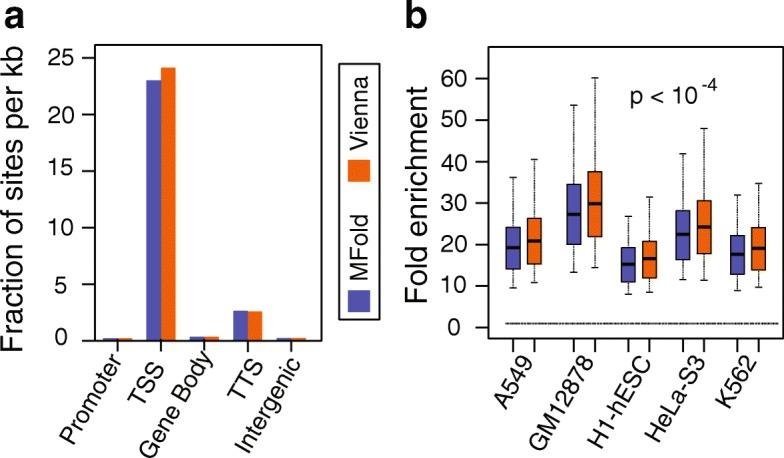
Significantly higher presence of highly stable DNA secondary structures at the TSS (± 250 nt) and co-localized with Pol II. **a** Gene location of highly stable secondary sites is plotted. Genomic regions were defined as described in “Methods”. The numbers of peaks were normalized by the total number of sites and the total size of genomic regions. **b** Null model analysis of the intersection of highly stable secondary structure sites with Pol II ChIP-seq peaks demonstrates enrichment patterns in five cell lines (*p* < 10^− 4^, permutation analysis). Fold enrichment is defined as the ratio of the actual number of intersecting regions over the mean of the number of intersections of 10,000 instances of randomly shuffled secondary structure sites. Solid line at fold enrichment = 1 corresponds to no change

To determine the extent of DNA secondary structure formation potential from single-stranded DNA on a genome-wide level, we used the Mfold program [33] and the ViennaRNA program [34] with thermodynamic parameters for DNA analysis to calculate the free energy of DNA secondary structure formation along the entire human genome sequence. For an input sequence, these programs apply algorithms that search the space of secondary structures of single-stranded DNA and estimate a corresponding free energy value. A more negative free energy value indicates a more stable DNA secondary structure. Therefore, the most favorable structure and free energy value were selected for each sequence to represent local propensity to form DNA secondary structures. Both programs can predict stem-loop structures, and, in addition, the ViennaRNA program allows for the prediction of quadruplex formation. To cover the human genomic sequence (build GRCh37/hg19), we divided the entire human genome into sliding windows of 300 nt, with a step size of 150 nt. For a total of 19,945,463 segments analyzed, the average free energy of DNA secondary structure formation calculated by Mfold and ViennaRNA was − 26.8 ± 9.5 kcal/mol and − 30.2 ± 11.9 kcal/mol, respectively (Additional file 1: Table S1). The range of values predicted by the two programs was − 180.5 to 8.1 kcal/mol for Mfold and − 937.4 to 0 kcal/mol for ViennaRNA, suggesting a highly variable propensity for DNA secondary structure formation genome-wide. Globally, free energies reported by the two programs are highly correlated (cor = 0.911, *p* < 10^− 16^); however, there are some differences mostly due to the presence of G-quadruplexes found by ViennaRNA. Notably, 1,350,698 windows (7%) contain G-quadruplexes as a part (~ 5% of nucleotides within these windows) of the overall predicted structure. Using ViennaRNA, windows with and without G-quadruplexes have a mean free energy of − 48 and − 29 kcal/mol, respectively, and the difference in free energies is statistically significant (*p* ≈ 0; Mann–Whitney U Test). However, differences between predicted free energies using ViennaRNA with and without G-quadruplexes in the same window tend to be relatively small (median difference = − 8.4 kcal/mol). Previous work has identified thresholds of predicted free energy for DNA secondary structure formation wherein the structure has the ability to interfere with cellular processes [40, 41]. Using the same threshold, we defined sites of highly stable DNA secondary structure as having at least seven consecutive windows with a free energy value in the top 5% most stable structures predicted across the genome (below − 43.6 kcal/mol for Mfold or − 51.7 kcal/mol for ViennaRNA). We identified 15,826 Mfold sites and 14,778 ViennaRNA sites, with sizes ranging from a minimum of 1200 nt up to over 20,000 nt in length, but in total making up less than 1% of the genome (Additional file 1: Table S1). The 74% of the Mfold sites are found overlapping with gene bodies (Additional file 1: Table S2; “Methods” for details), with 4419 out of 15,826 (28%) sites coinciding with a TSS ± 250 nt. Similarly, 76% of the ViennaRNA sites overlap with a gene body, including 4326 out of 14,778 (29%) sites coinciding with the TSS ± 250 nt. When normalized to the total size of each class of genomic regions (i.e., TSS, gene body, etc.), it becomes evident that highly stable secondary structure sites are greatly enriched at TSSs (Fig. 1a, Additional file 1: Table S2; p ≈ 0, Fisher’s exact test). These results demonstrate that predicted DNA secondary structure formation is widespread across the human genome, and the location of potential highly stable DNA secondary structure regions suggests that these structures could participate in gene transcription.

Because of the high co-localization with TSSs genome-wide, we next determined whether potential highly stable DNA secondary structure sites are enriched with the binding sites of Pol II, using Pol II ChIP-seq data from the encyclopedia of DNA elements (ENCODE) in five cell lines: A549, GM12878, H1-hESC, HeLa-S3, and K562. The level of enrichment for the association was measured by the fold enrichment of Pol II ChIP peaks intersecting highly stable secondary structure sites in comparison to that of random sites (see “Methods” for details). In all five cell lines and for both Mfold and ViennaRNA, we found a 15- to 30-fold enrichment of DNA secondary structure sites at peak sites of RNA Pol II binding (Fig. 1b). These results demonstrate that sites of predicted highly stable DNA secondary structure are significantly enriched at the Pol II binding sites.

To measure the level of enrichment for RNA Pol II ChIP-seq coverage at sites of predicted highly stable DNA secondary structure, we quantified the number of ChIP-seq reads at each Mfold or ViennaRNA site and compared this value to the mean number from randomly shuffled reads (see “Methods”). We find that the degree of significant enrichment is similar to that of the association seen using the Pol II ChIP-seq peaks (Additional file 1: Figure S1). The Mfold and ViennaRNA sites were enriched for RNA Pol II coverage in all five cell lines with an average enrichment of approximately 50- to 100-fold. Overall, these results suggest that alternative DNA secondary structure formation could influence the active transcription mechanism on a genome-wide level.

### The promoter-proximal regions of genes with paused RNA polymerase II are able to form highly stable DNA secondary structures

RNA Pol II pausing is a very common step in the transition from initiating to elongating transcription, resulting in a paused polymerase downstream of the TSS and participating in transcription regulation [16–18, 20, 21]. Results described above strongly suggest that stable secondary structure formed by single-stranded DNA during transcription could play a significant role in Pol II pausing. To define genes in the paused states, we determined the RNA Pol II traveling ratio (TR) as previously described [22]. In brief, we first stratify all RefSeq genes into the Pol II-bound and the no-Pol II groups, and for the Pol II-bound group, the TR was calculated as a ratio of coverage density at a region between − 30 nt to + 300 nt of the TSS over coverage density across the rest of the gene body. Using the same Pol II ChIP-seq datasets as in our association study, we found that 86 and 84% of Pol II-bound genes in HeLa-S3 and H1-hES cells, respectively, are paused, as defined by a TR > 2 (Additional file 1: Figure S2A and Table S3), similar to previous studies [22]. Next, we determined whether the regions surrounding the TSS of paused genes (PAU) are more prone to DNA secondary structure formation than non-paused genes (NPA) or those with no Pol II (NP2). Using our genome-wide Mfold data, we found that the region proximal to the TSS (250 nt upstream to 250 nt downstream) of paused genes displays a significantly lower ΔG than non-paused genes or genes without Pol II bound in all investigated cell lines (Additional file 1: Figure S2B and Table S4), further suggesting the potential for alternative DNA secondary structures to contribute to Pol II pausing in human cells. Very similar results were obtained using ViennaRNA sites (data not shown).

**Fig. 2:**
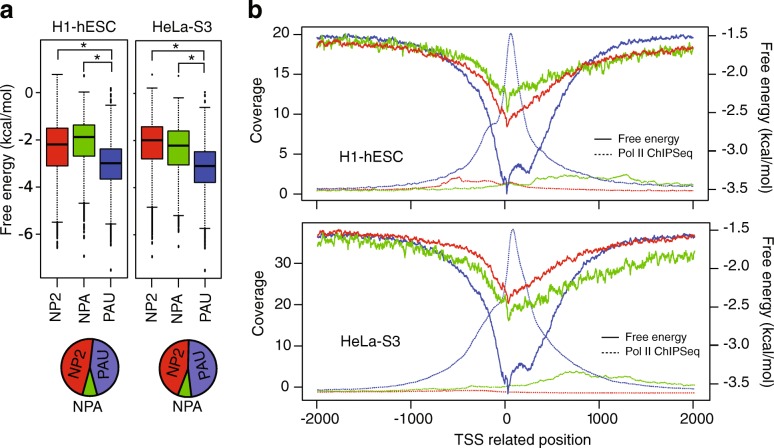
Highly stable DNA secondary structures preferentially form at the promoter-proximal regions of paused genes. **a** Box plots show the mean free energy at the TSS ± 250 nt in two cell lines: H1-hESC (*left*) and HeLa-S3 (*right*). Based on the traveling ratio, genes were classified as no Pol II (*NP2*, *red*), non-paused (*NPA*, *green*), and paused (*PAU*, *blue*). Numbers of each group of genes in each cell line (shown in Table S3) are depicted in pie charts. *p < 2.2 × 10^− 16^, *t*-test (Additional file 1: Table S4). **b** Average free energy profiles (*solid line*) and average Pol II ChIP-seq coverage profiles (*dotted line*) are shown in two cell lines: H1-hESC (*top*) and HeLa-S3 (*bottom*). Paused, non-paused, and no Pol II genes are shown in *blue*, *green*, and *red*, respectively. The Mfold analysis was used here, and the ViennaRNA anlysis is shown in Additional file 1: Figure S2D

To better recapitulate the potential for DNA single-strandedness during gene transcription, we next calculated the free energy of secondary structure formation by Mfold and ViennaRNA, including G-quadrulex prediction for the same TSS proximal regions, but applying a 30-nt sliding window with a 1-nt step size to the non-template strand sequences of the regions. This window size better represents the estimated size of the transcription bubble, during which the double-stranded DNA is unwound to allow transcription to occur. The 1-nt step size provides the highest possible resolution of secondary structure free energy estimation. Upon unwinding of the double-stranded DNA, the non-template strands are more likely to form DNA secondary structure than template strands, which are occupied by nascent RNA and extensive interactions with the Pol II complex. Notably, high resolution secondary structure ΔG spanning TSS ± 250 nt reveals similar features to that of low resolution data in all six investigated cell lines (Fig. 2a and Additional file 1: Figure S2C contain Mfold data; Additional file 1: Table S4 contains data from both Mfold and ViennaRNA data), in which the TSS ± 250 nt regions of paused genes shows a significantly lower ΔG than non-paused genes or genes without Pol II bound. Also, the free energies by these two programs are highly correlated at the TSS ± 250 nt regions (Additional file 1: Table S4; cor = 0.999, *p* < 0.02 in six cell lines).

We next sought to determine the location of highly stable secondary structures relative to the Pol II binding site. Regions spanning from 2000 nt upstream to 2000 nt downstream of each gene were analyzed (TSS proximal regions), and average plots of ΔG were shown for paused genes (PAU), non-paused genes (NPA), and genes not bound by Pol II (NP2) (Fig. 2b contains Mfold data; Additional file 1: Figure S2D contains Vienna RNA data). For paused genes, the most stable DNA secondary structures occur at regions directly surrounding and slightly downstream of the TSS, as indicated by the sharp drop in relative free energy. These effects are diminished in non-paused genes and genes without Pol II binding. Notably, the average-gene free energy minimum is slightly upstream of the average-gene Pol II peak at paused genes.

Interestingly, when we compare the free energy landscape between template and non-template strands of the same DNA region, we found a strand bias around the TSS of paused genes, but not of non-paused genes (Additional file 1: Figure S3A). The non-template strand has a significantly higher propensity to form DNA secondary structures than the template strand (*p* = 1.9 × 10^− 28^, *t*-test for average free energy at the − 30 to + 300 region from the TSS) (Additional file 1: Figure S3B).

Together, these data demonstrate that the TSS-proximal region of genes with Pol II pausing are more likely to form DNA secondary structures than those genes that are not paused or are not bound by Pol II, suggesting a role for DNA secondary structures in promoter-proximal RNA Pol II pausing.

### Promoter-proximal RNA polymerase II pausing occurs at sites of stable DNA secondary structure formation genome-wide

Because Pol II ChIP-seq is based on immunoprecipitation of RNA Pol II, and its signals might not recapitulate all actual pausing sites, but rather loci bound by Pol II, we next performed our analysis with data from GRO-seq [35] and NET-seq [36]. Both techniques are designed specifically to sequence nascent RNA transcribed by RNA Pol II, therefore marking pausing sites. High resolution average coverage profiles of GRO-seq and NET-seq data from HeLa-S3 cells (Additional file 1: Figure S4A) show very similar patterns to that of the Pol II ChIP-seq profile, in which high levels of short nascent RNAs produced on the coding strands from the TSS co-localize with secondary structure free energy minima for paused genes only.

**Fig. 3:**
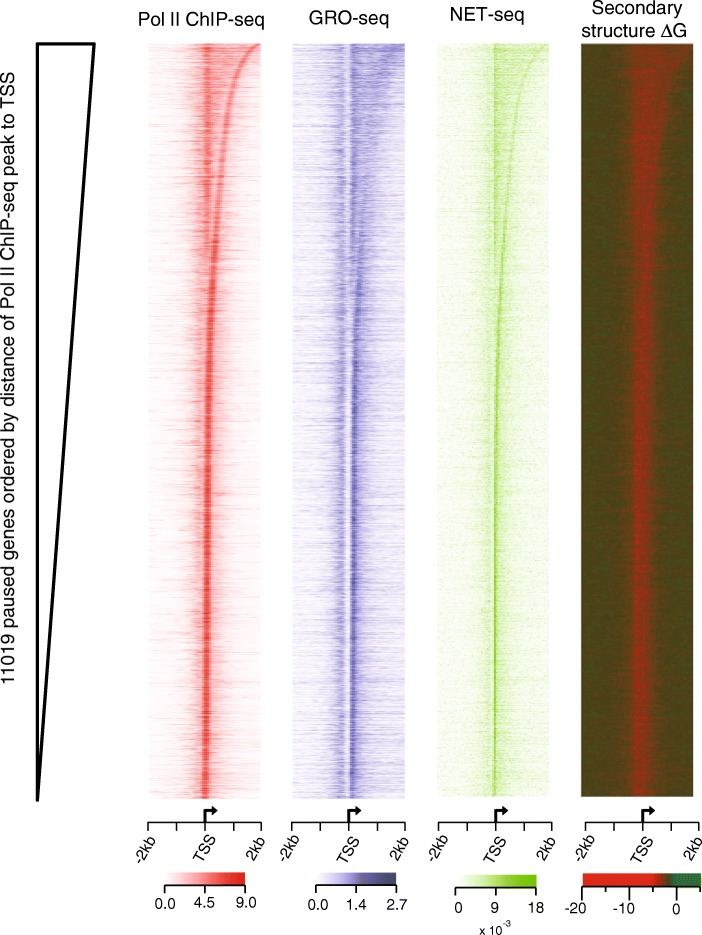
Pol II occupancy signals from Pol II ChIP-seq, GRO-seq, and NET-seq strongly correlate with stable DNA secondary structures of paused genes. Heat map representations of Pol II ChIP-seq (*red*), GRO-seq (*blue*), NET-seq (*green*) coverage and free energy (*green-red*) profile in HeLa-S3 cells are shown at the TSS ± 2 knt region. Paused genes (*n* = 11,019) were ordered by the distance of the Pol II ChIP-seq peak summit from each gene’s TSS

To exclude the possibility that these average profiles could be obscured by a relatively small group of genes with extremely high Pol II or low free energy profiles, we analyzed and displayed all genes using heat maps. For each paused gene (*n* = 11,019), the peak position of Pol II ChIP-seq within the TSS proximal regions (TSS ± 2 knt) was first identified, and then genes were ordered by the distance between the peak summit and its nearby TSS as shown in Fig. 3. We found that a distinct pattern of Pol II ChIP-seq peak summits is recapitulated by the GRO-seq and NET-seq data. Most importantly, the distribution of distances of Pol II ChIP-seq, GRO-seq, and NET-seq summits relative to TSSs corresponds well with that of free energy minima (i.e., sites of highly stable secondary structure).

Interestingly, approximately 37% of non-paused genes (*n* = 655) also contain Pol II ChIP-seq peaks within TSS proximal regions, and these peaks are located more than 300 nt downstream of the TSS (therefore, by the TR definition, they were classified as non-paused genes). When analyzed based on Pol II ChIP-seq peak summits, these non-paused genes show a similar, close relationship between Pol II pausing and the formation of stable DNA secondary structures (Additional file 1: Figure S4B), even in the absence of Pol II signals directly at TSSs.

These data demonstrate that Pol II-pausing signals are very close to and track sites that have high potential to form relatively stable DNA secondary structures of single-stranded DNA.

### Pausing sites defined by mNET-seq spikes are preceded by highly stable DNA secondary structures

**Fig. 4:**
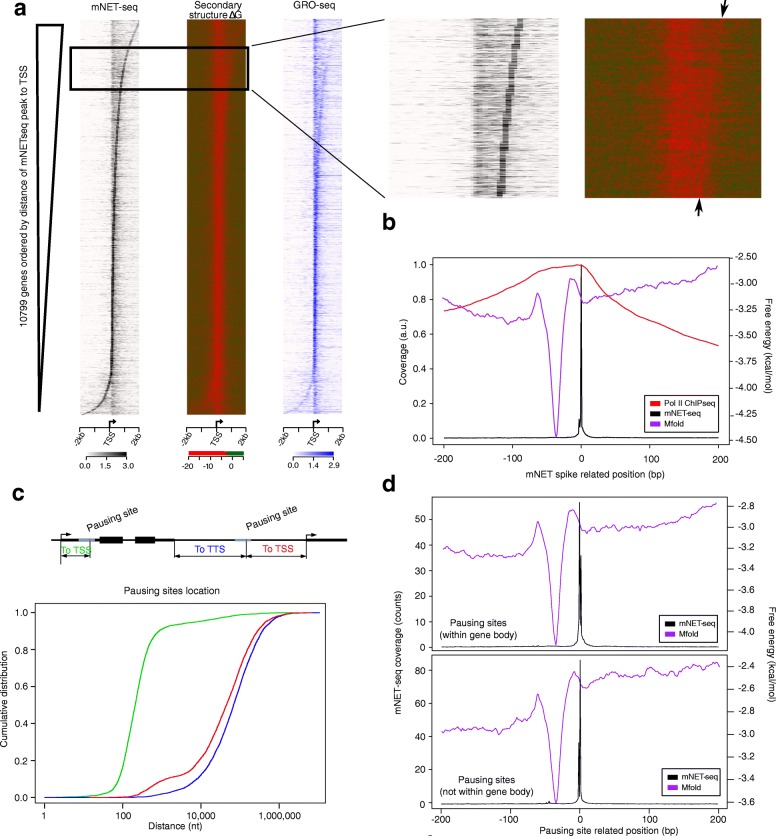
Single-nucleotide resolution signals of mNET-seq demonstrate highly stable secondary structures located upstream of the paused sites. **a** Heat map representations of mNET-seq coverage (*black*), secondary structure free energy (*green-red*), and GRO-seq coverage (*blue*) for Pol II bound genes in HeLa-S3 cells are shown. Pol II-bound genes were ordered by the distance of the strongest mNET-seq read spike to each gene’s TSS. A high magnification of a section of the heat maps displays a thin, bright line of relatively low secondary structure free energies (marked by *arrows*). **b** An average free energy profile (*purple*) and average Pol II coverage (*red*) centered at the strongest mNET-seq read spike (*black*) are shown (*n* = 10,428). At the average free energy of − 3.25 kcal/mol, DNA secondary structures are, on average, about 10 to 40 nt upstream of the peak of the highest mNET-seq read spikes. **c** Schematic representation of genic and intergenic pausing sites relative to annotated gene transcription start and termination sites. Cumulative distribution of distances between pausing sites to the TSS or TTS: pausing sites within human RefSeq annotated genes (to TSS, *green*), and pausing sites located outside of human annotated genes (to TSS (*red*) and to TTS (*blue*)). **d** Average profile plot of mNET-seq coverage (*black*) and secondary structure free energy (*purple*) at Pol II pausing sites that are located within annotated human genes and centered on the highest mNET-seq read spike are shown (*top panel*). At the average free energy of − 3.2 kcal/mol, DNA secondary structures are, on average, about 24 to 48 nt upstream of the peak of the highest mNET-seq read spikes. Average profile plot of mNET-seq coverage (*black*) and secondary structure free energy (*purple*) at Pol II pausing sites that are not located within annotated human genes and centered on the highest mNET-seq read spike are shown. At the average free energy of − 2.8 kcal/mol, DNA secondary structures are, on average, about 24 to 52 nt upstream of the peak of the highest mNET-seq read spikes (*bottom panel*). The Mfold analysis was used here, and the ViennaRNA anlysis is shown in Additional file 1: Figure S5

We have shown that Pol II pausing sites are close to sites that have high potential to form stable DNA secondary structures. We next investigated whether highly stable DNA secondary structures are more likely to form upstream of Pol II pausing sites as indicated by average gene plots (Fig. 2b), to better understand how DNA secondary structures influence Pol II pausing. Using high quality mNET-seq data [37], which contains single-nucleotide resolution genome-wide sequence data of nascent RNA for each Pol II-bound gene in HeLa-S3 cells, we first identified the nucleotide where the highest mNET-seq read spike is, and then ordered all loci by the distance between the mNET-seq spike position and the TSS. Figure 4a shows that the mNET-seq read spikes of all Pol II-bound genes are closely localized with highly stable DNA secondary structures, recapitulating what we found in Fig. 3 with Pol II ChIP-seq peak signals. The red line representing highly stable secondary structures not only closely follows the mNET-seq spike pattern, but is also sharper (Fig. 4a) than the corresponding line obtained with Pol II ChIP-seq-based ordering (Fig. 3). This suggests that stable DNA secondary structure formation has a stronger and more precise correlation with the mNET-seq read spikes, and that mNET-seq read spikes can represent pausing sites more accurately. Next, to determine the position of secondary structure formation relative to Pol II pausing sites, we plotted the average profiles of the highest mNET-seq read spikes, in a range of ± 200 nt from each mNET-seq read spike position, and compared them to an average plot of secondary structure formation ΔG (Fig. 4b). We observe a sharp free energy minimum about 10 to 40 nt upstream of the mNET-seq spikes. Notably, this minimum is not only sharp but also deep, with the lowest mean free energy having a value (− 3.30 kcal/mol) that is in the 15th percentile of free energies of all TSS-proximal regions. Inclusion of G-quadruplexes did not change the shape or position of the sharp free energy minimum relative to the mNET-seq peaks, but it did lower the ΔG of the overall average curve by − 0.5 kcal/mol (Additional file 1: Figure S5A). In addition to the average plots, the secondary structure-forming free energies are also individually plotted for each pausing site (Additional file 1: Figure S6). The plot shows a very similar pattern to the averaged plot of Fig. 4b, in which a deep free energy minimum is present just upstream of the pausing sites. All of these findings strongly suggest that highly stable DNA secondary structures formed within 50 nt upstream of pausing sites are a common feature of Pol II pausing.

Because both paused (Fig. 3) and non-paused genes (Additional file 1: Figure S4B) show the same strong correlation with stable secondary structure in our analysis of RefSeq annotated genes, we next wanted to refine pausing site loci in an unbiased manner (i.e., not based on annotated genes). We hypothesized that pausing sites produce a strong Pol II pausing-related signature evident in all pausing-relevant data sets. Specifically, we first used GRO-seq peaks (*n* = 37,276) that intersect significantly (>  50% overlap) with Pol II ChIP-seq peaks (*n* = 26,487). For the resulting list (*n* = 15,470) of genomic regions, we further required that each peak have mNET-seq coverage above background (5% FDR, *n* = 13,931). We then ranked all pausing loci from 1 (the strongest) to 13,931 (the weakest) based on the sum of each ranked signal intensity: Pol II ChIP-seq, GRO-seq, NET-seq, and mNET-seq (Additional file 1: Figure S7A).

This analysis resulted in a ranked list of 13,931 genomic regions that we identify as Pol II pausing sites, with 57% of them (*n* = 7972, Additional file 2) located within RefSeq gene bodies. When the distances of these sites to TSSs were measured (Fig. 4c), these pausing sites within gene bodies were located about 100 nt downstream of TSSs (Fig. 4c, green line), and a sharp peak of average free energy of stable secondary structure precedes the pausing site about 24 to 48 nt upstream at a free energy of − 3.2 kcal/mol (Fig. 4d, top panel). Three genes, *SNAI3-AS1*, *DHX8*, and *SMC5*, ranked as 7, 239, and 486, respectively, are shown in Additional file 1: Figure S7B as having highly stable DNA secondary structures located immediately upstream of the highest mNET-seq spikes.

The remaining 43% of pausing loci (*n* = 5959, Additional file 3) are located in either intergenic regions (67%) or enhancer/promoter regions of genes (33%). These pausing sites are located much further away from the nearest TSS or transcription termination site (TTS) of genes (i.e., either downstream or upstream) (Fig. 4c). Most strikingly, the average ΔG of secondary structure formation for those loci (about 24 to 52 nt upstream at a free energy of − 2.8 kcal/mol; Fig. 4d, bottom panel) is very similar to that observed for intragenic pausing loci. Again, inclusion of G-quadruplexes left the location and shape of the sharp free energy minimum unaffected, and lowered the average free energy at the minimum by − 0.4 and − 0.2 kcal/mol for intragenic and intergenic loci, respectively (Additional file 1: Figure S5B). In addition to the average plots, for both groups, the secondary structure-forming free energies are also individually plotted for each pausing site (Additional file 1: Figure S8A, B), and both plots show the same pattern as the averaged plots in Fig. 4d, in which a deep free energy minimum is present 10 to 50 nt upstream of the pausing sites. Further, we plotted the frequency of the positions of secondary structures with free energies within the lowest 2% of free energy values (most stable structures) and found that the structures with the lowest free energies are also enriched just upstream of the pausing sites (Additional file 1: Figure S8C). Conversely, there is a slight depletion in the highest 2% free energy distribution (unfavorable to form the structures) at the region just upstream of the pausing sites, and a uniform density from structures with middle values. These findings further support the close relationship between Pol II pausing and the propensity to create highly stable DNA secondary structures upstream of pausing loci, and the same stable DNA secondary structure signature is observed beyond the RefSeq annotated genes*.*

### Alternative DNA secondary structures identified by probing experiments are enriched at the promoter-proximal regions of paused genes in human cells

**Fig. 5:**
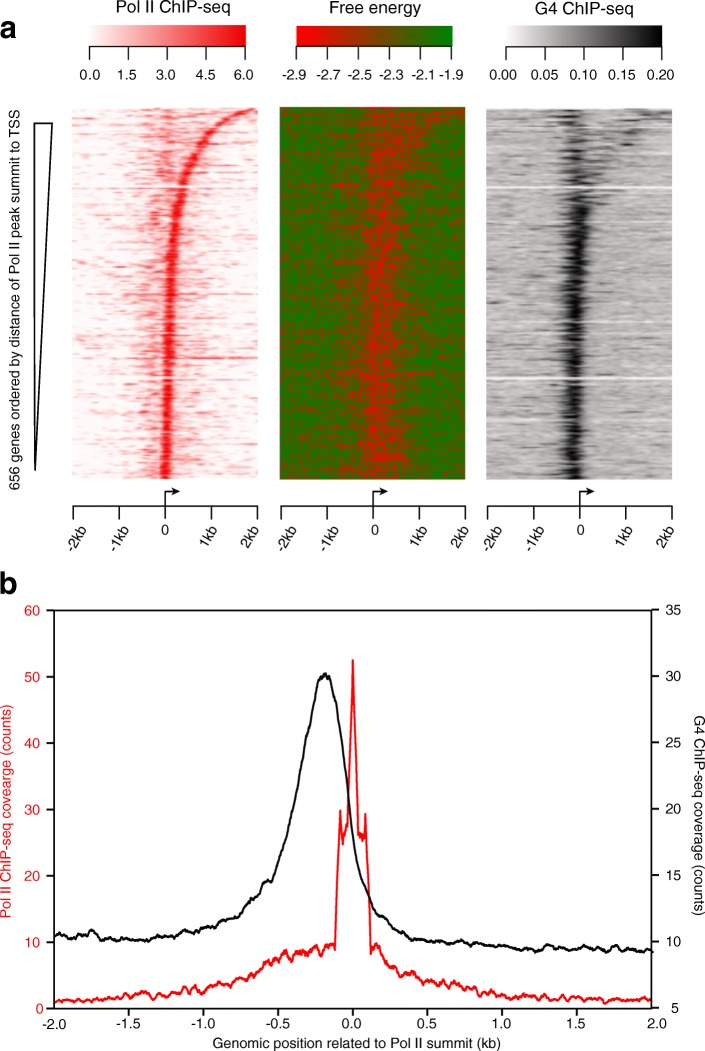
G4 structures identified by G4-specific antibody in NHEK cells strongly correlate with Pol II pausing sites and free energy minima of computed structures. **a** Heat map representations of Pol II ChIP-seq (*red*), and G4 ChIP-seq (*black*) coverage and free energy (*green-red*) profile in NHEK cells are shown at the TSS ± 2 knt region. Genes with G4 structures within the TSS + 2 kb region (*n* = 656) were ordered by the distance of the Pol II ChIP-seq peak summit from each gene’s TSS. **b** Average G4 structure profiles (*black line*) and average Pol II ChIP-seq coverage profiles (*red line*) are generated from the data described in **a**

Next, we investigated whether alternative DNA secondary structures can be detected at the promoter-proximal regions of paused genes in human cells. The existence of G-quadruplexes (G4), a subtype of alternative secondary structures, has been experimentally validated in NHEK cells, using an antibody specific to G4 structures [38]. To determine whether G4 structures are located directly upstream of paused Pol II, we identified 656 genes with G-quadruplex structures within their TSS + 2 kb region, and 94% of them are paused in NHEK cells (TR > 2). Next, we displayed all 656 genes using heat maps to analyze the relationship of the location of Pol II, secondary structure free energy minima, and G4 structures (in which genes were ordered by the distance between the Pol II peak summit and its nearby TSS as in Fig. 3). We found that the distribution of G4 structures corresponds well with that of Pol II and free energy minima (Fig. 5a), indicating the presence of G4 structures at paused Pol II sites, thereby validating the computational secondary structure predictions. The average coverage profile (Fig. 5b) shows the enrichment of G4 structures immediately upstream of paused Pol II, in qualitative agreement with the location of predicted free energy minima relative to mNET-seq spikes (Fig. 4). One important note is that both the Pol II ChIP-seq and G4 ChIP-seq data are not at single-nucleotide resolution as is the case for the mNET-seq data; therefore, the distance between the peaks of the Pol II and G4 average plots is not an accurate estimate of the characteristic distance between a G4 structure and paused Pol II.

**Fig. 6:**
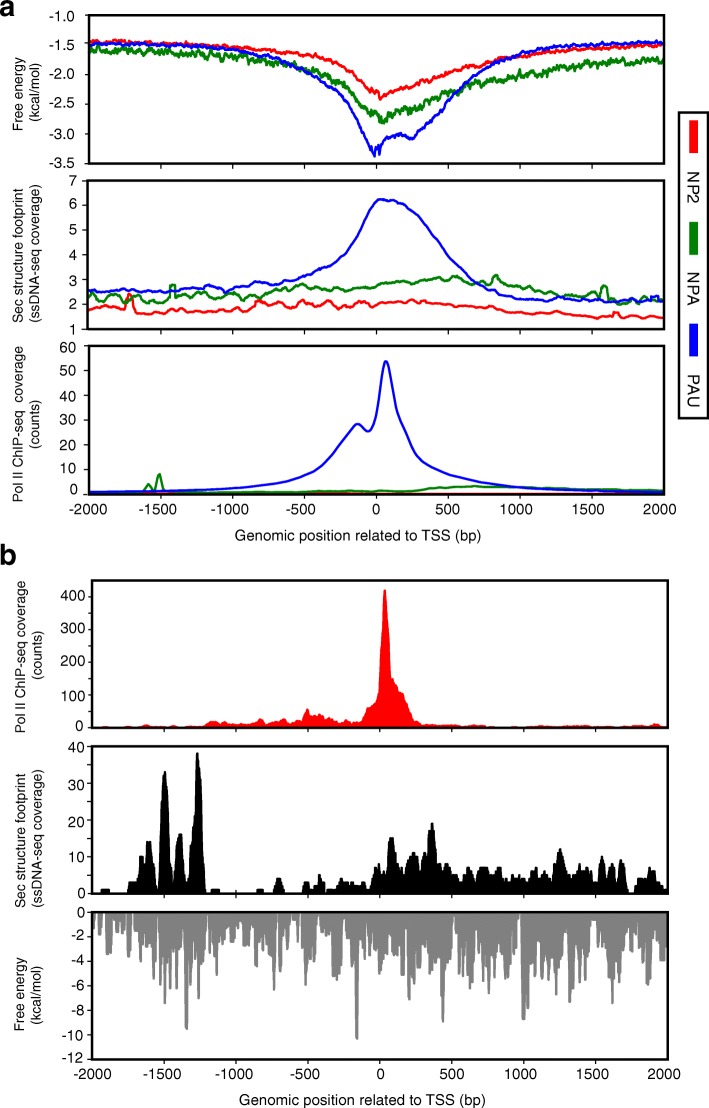
Stable DNA secondary structures detected in Raji cells preferentially form at the promoter-proximal regions of paused genes. Based on the traveling ratio, genes in Raji cells were classified as no Pol II (*NP2*, *red*), non-paused (*NPA*, *green*), and paused (*PAU*, *blue*). Numbers of each group of genes are listed in Table S3. **a** Average free energy profiles (*top*), in vivo secondary structure footprints (*middle*), and average Pol II ChIP-seq coverage profiles (*bottom*) are shown. Heat map representations of the same data are shown in Additional file 1: Figure S8. **b** Profiles of Pol II ChIP-seq (*top*), in vivo secondary structure footprints (*middle*) and the predicted free energy (*bottom*) at TSS ± 2 kb of human heat shock gene (*HSPA1B*) are shown

Genome-wide non-B DNA secondary structures have been recently determined in Raji cells using the combination of permanganate footprinting, high-throughput sequencing, and the sequence motifs of non-B DNA [39]. A total of 10,444 non-B DNA structures were shown to overlap with RNA Pol II binding sites [39]. We then examined how these structures are distributed among genes with different Pol II pausing status at the TSS ± 2 kb region. Among PAU, NPA, and NP2 genes in Raji cells, again there is a difference in the propensity to form secondary structures calculated by computational analysis, as we observed in other cells, with paused genes having lower free energy minima than the other two groups (Fig. 6a, top panel). More importantly, only the paused gene group displays a peak of secondary structure footprinting signals just downstream of the TSS (Fig. 6a, middle panel), which match very well with the distribution of the average predicted free energy profiles. Taken together with the presence of Pol II peaks in paused genes only (Fig. 6a, bottom panel) this analysis suggests that the detected secondary structure in Raji cells could contribute to Pol II pausing. To exclude the possibility that these results are driven by outliers, we also plotted heat maps, showing a strong correlation among the secondary structure footprints, the predicted low free energies, and the location of Pol II (Additional file 1: Figure S9). Three examples of paused genes, *HSPA1B* (Fig. 6b), *MYC*, and *CDK4* (Additional file 1: Figure S10), are shown with the profiles of Pol II, the secondary structure footprints, and the predicted free energy, to display this association at specific loci. These two experimental structural analyses provide direct evidence of the existence of DNA secondary structures in the regions upstream of promoter-proximal pause sites and support the possible presence of DNA secondary structures at these loci as predicted by their low free energies, thereby validating the use of calculated free energies in our analyses.

We noticed that the majority (80 to 90%) of Pol II-bound genes are paused in all eight investigated cell lines (Additional file 1: Table S3). Importantly, this was based on the stratification of genes according to traveling ratios (TRs) for the three groups (NP2, NPA, and PAU) without accounting for differences or similarity of gene expression across the cell lines. Because a significant amount of DNA sequences are shared among different cell types, this prompted us to investigate pausing states of genes across these eight cell lines derived from different cell types. If Pol II pausing can be driven by DNA sequences/structures, strong overlaps of genes with similar pausing states among these different cell lines will be observed. Indeed, gene ranks of TR-based pausing states for all Pol II-bound genes are highly correlated among cell lines (Additional file 1: Figure S11A). Spearman correlation coefficients calculated for all pairs of cell lines range from 0.71 between H1-hESC and NHEK to 0.88 between GM12878 and K562. Furthermore, a heat map of pausing states across these cell lines (Additional file 1: Figure S11B) clearly demonstrates consistent gene states across cell lines with genes in either paused or non-paused states. Moreover, we identified the numbers of genes which switch pausing states between cell lines: from paused to non-paused and from non-paused to paused in each pair of cell lines (Additional file 1: Figure S11C). The “switched” genes in each cell line pair are about 1–3% of total RefSeq genes, and TRs of those genes are rather low (80% have a TR within the first quartile of all TRs). These differences may result from the threshold used to group genes into paused and not paused categories, and also may be due to variable quality of Pol II ChIP-seq experiments across different laboratories. It is also possible that cell type-specific factors which contribute to Pol II pausing are present.

These analyses suggest that the degree of Pol II pausing and the genes at which Pol II pauses are similar and shared across eight different human cell lines, and that DNA secondary structure formation detected in NHEK and Raji cells plays a role in the ubiquitous process of Pol II pausing. Importantly, these analyses and results provide a concrete mechanism—propensity to form DNA secondary structures—for how high GC content at TSSs influences Pol II pausing.

### DNA secondary structures affect RNA pol II promoter-proximal pausing in vitro

**Fig. 7:**
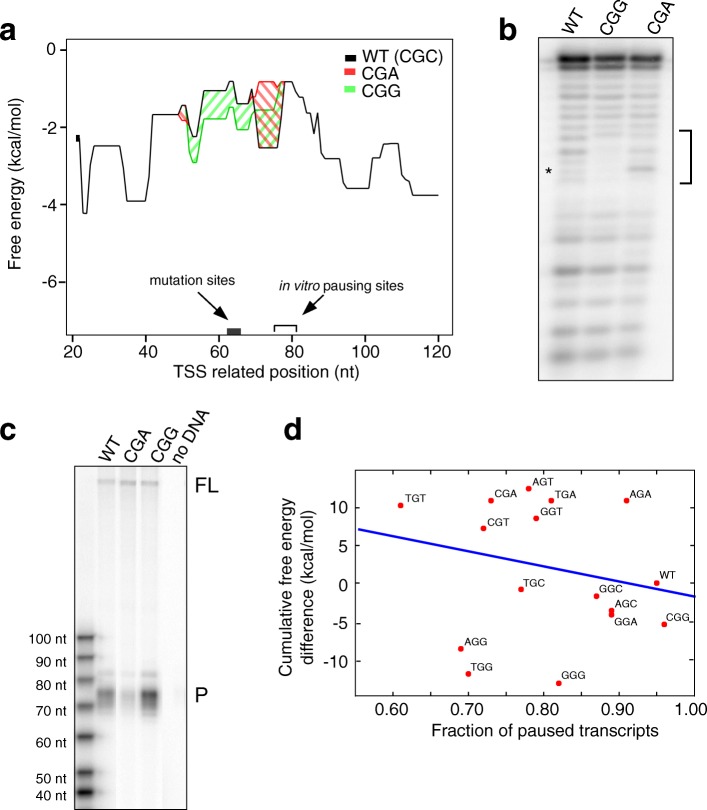
*HSPA1B* mutants affecting DNA secondary structure formation demonstrate differential pausing in vitro*.* **a** Plot shows free energy profile of *HSPA1B* gene around the in vitro pausing site. The difference in free energy (ΔG) between CGA mutant (less stable structure) and WT (CGC) is marked in *red*, while the **Δ**G difference between CGG (more stable structure) and WT is marked in *green*. Mutated sites are highlighted. In vitro transcription conditions (60 mM KCl, 7 mM MgCl_2_, and 30 °C) were used for Mfold analysis. **b** Structural probing of WT (CGC), CGG, and CGA with mung bean nuclease under in vitro transcription conditions. Each of the DNAs (30 nt) were treated with mung bean nuclease, which specifically cleaves single-stranded regions, and the products resolved on denaturing polyacrylamide gels. It showed that the CGG mutant displayed more protection at the region around the mutation (the affected region marked by a *bracket* and the mutation site marked by an *asterisk*), indicating the presence of double-stranded regions and thus the formation of hairpin structure. In contrast, the CGA mutant was more susceptible to the mung bean nuclease cleavage at the mutation site, suggesting the destabilization of the hairpin. **c** A representative polyacrylamide gel shows in vitro transcription of full-length (*FL*, 311-nt runoff transcripts) and paused (*P*) transcripts of wild-type *HSPA1B*, CGA, and CGG mutants, and no DNA template control in HeLa nuclear extracts. **d** The dependence of cumulative free energy difference and experimentally measured fraction of paused transcripts is shown. The cumulative free energy difference for each variant is determined from all possible secondary structures affected by the mutations (Additional file 1: Table S5). The *line* generated using robust linear regression (RLM function in Python) shows a correlation of strong RNA Pol II pausing associated with stable DNA secondary structures

To directly test whether alternative DNA secondary structure could contribute to Pol II pausing, we performed in vitro transcription experiments using the early promoter region of the human *HSPA1B* gene. RNA Pol II promoter-proximal pausing of the *HSPA1B* gene has been well characterized. Bunch et al. [42] used a single-stranded DNA fragment of *HSPA1B* as bait to identify a pausing factor TRIM28 in human cells, and showed that the non-template DNA sequence of *HSPA1B* is critical for TRIM28 binding, especially the region from 50 to 80 nt downstream of the TSS, and that the Pol II pausing sites is centered around nucleotide + 75. Our correlation data showed that a highly stable secondary structure is likely to form about 10 to 50 nt upstream of a Pol II pausing site. Therefore, within nucleotides + 50 to + 65, we changed only two nucleotides at 62 and 64 nt downstream of the TSS (Additional file 1: Figure S12A) in order to minimize the probability of disturbing transcription in general but maximize the effect on DNA secondary structure formation (Fig. 7a). Using an unbiased approach, we generated 16 sequence variants, including wild type (WT), with either more stable or disrupted secondary structures compared to the WT, based on the non-template strand sequences. In Fig. 7a, the free energy profiles of two such mutants are shown: in various sequence windows (30 nt each), the CGG mutant forms more stable secondary structures and the CGA mutant destabilizes the structures. Structural probing analysis of one window sequence using mung bean nuclease (Fig. 7b), which specifically cleaves single-stranded regions, showed that the CGG mutant displayed more protection at the region around the mutation, indicating the presence of double-stranded regions and thus the formation of hairpin structure. In contrast, the CGA mutant was more susceptible to the mung bean nuclease cleavage at the mutation site, suggesting the destabilization of the hairpin. Using an in vitro transcription assay (Fig. 7c), Pol II pauses within the *HSPA1B* wild-type sequence, indicated by a buildup of transcripts centered around 75 nt in length, as described in a previous study [42]. The CGG mutant, which stabilized the formation of secondary structure, displayed stronger pausing signals than WT, while the CGA mutant, forming a less stable secondary structure, showed weaker pausing signals. For all 16 mutants, we quantified Pol II pausing using the ratio of the intensity of the paused signals to that of all transcripts, paused and full-length (311 nt runoff transcripts; Additional file 1: Table S5), and compared this ratio to the cumulative free energy difference between the mutant and the WT, which serves as an indicator for overall DNA secondary structure formation potential. Robust linear regression analysis (MM method, Huber’s weighting, RLM function from the statmodels package in Python) showed that the Pol II pausing ratio is anti-correlated with DNA secondary structure stability, with strong pausing associated with more stable DNA secondary structures (Fig. 7d). Further, when we analyzed the same data using free energy differences from template strand sequences (Additional file 1: Figure S12B), we found that the resulting slope from the regression analysis is − 20 and − 0.27 for the non-template and template strand, respectively, indicating that the free energy of structure formation from the non-template strand is more strongly anti-correlated with the experimental Pol II pausing data. These results support the role of DNA secondary structure in RNA Pol II promoter-proximal pausing and suggest that the secondary structure of DNA formed on non-template strands can affect the level of RNA Pol II pausing during transcription.

## Discussion

In this study, using DNA secondary structure calculation programs, we have, for the first time, provided an energetic potential for secondary structure formation across the human genome in an unbiased manner. This analysis not only indicates genomic regions with the potential to form secondary structures, but also estimates the relative propensity for these regions to actually form such structures. We found that DNA secondary structures are prone to form from single-stranded DNA at the promoter-proximal region of genes displaying Pol II pausing shortly after transcription initiation, with the secondary structure on average located upstream of the Pol II pausing sites. This DNA secondary structure–pausing site relationship is also present in regions located outside of the RefSeq annotated genes, suggesting a common feature associated with Pol II pausing at loci of coding genes and non-coding, intergenic DNA. Further, the presence of DNA secondary structures at the promoter-proximal regions of paused genes can be confirmed in human cells. The mutation analysis of the *HSPA1B* gene region demonstrates that disruption of DNA secondary structures proximal to pausing sites reduces Pol II pausing in HeLa nuclear extracts in vitro.

**Fig. 8:**
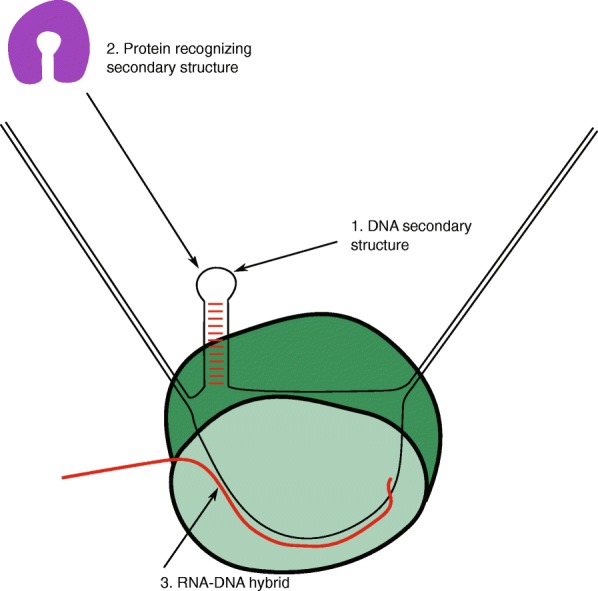
Possible mechanisms for DNA secondary structure mediation of promoter-proximal Pol II pausing. The transcription complex (*green*) could be paused via multiple mechanisms: (1) by a secondary structure formed on the non-template strand of DNA itself; (2) by a protein recognizing such a structure; or (3) by an RNA:DNA hybrid (*red* and *black lines*)

These structures have the ability to form when the DNA duplex is unwound during pioneering rounds of transcription. Additionally, secondary structures can form as a by-product of supercoiling upstream of gene regions, without the need for prior transcription of the structure-forming site. DNA secondary structure can potentially mediate promoter-proximal Pol II pausing through a number of mechanisms (Fig. 8). As shown for the proenkephalin gene [15], DNA structure in the promoter can serve as a binding site for proteins involved in transcriptional regulation, and possibly proteins involved in regulation of Pol II pausing. Recent work identified TRIM28 as a mediator of Pol II pause and release at the *HSPA1B* gene in human cells through its interaction with a single-stranded DNA sequence corresponding to nucleotides + 50 to + 80 (from the TSS) on the non-template strand of the gene, which may form stable secondary structure [42]. This raises the possibility that stable DNA secondary structure formation at the *HSPA1B* gene recruits TRIM28 to sites of Pol II pausing and mediates promoter-proximal Pol II pausing. The second possible mechanism for DNA secondary structure mediating Pol II pausing is through stabilizing the formation of the RNA:DNA hybrids. DNA secondary structure forming upstream of pausing sites on non-template strands could favor hybrid formation between RNA transcripts and the DNA templates, and temporarily create a barrier to DNA:DNA annealing at the 5′ end of the RNA:DNA hybrids, both of which may facilitate Pol II pausing. Our analysis shows an enrichment of stable RNA:DNA hybrids at the region of free energy minimum, just upstream of the pausing sites within RefSeq annotated genes (Additional file 1: Figure S13). The formation of stable DNA secondary structures on the non-template strand and the formation of RNA:DNA hybrids can occur at the same location within transcription bubbles at the same time. In fact, it is energetically favorable when both structures are formed. R-loop structures can cause significant stalling of polymerase in vitro [43], and conditions which stabilize or destabilize R-loops have been shown to increase or decrease the stall, respectively [44, 45]. Also, studies have shown that R-loop accumulation is associated with Pol II pausing in human breast cancer cell lines [29, 46]. We analyzed the position of R-loops relative to the pausing sites using genome-wide DRIPc-seq data from the human Ntera 2 cell line [47] and did not observe an accumulation of R-loops in the region just upstream of the pausing sites, as seen with the potential secondary structure formation (Additional file 1: Figure S14). It is important to point out that in these studies, the R-loop read peaks have a median size of 1.5 kb, and no individual R-loop footprints are available to compare to the location of pausing sites at the same scale. Further, the crystal structure of the Pol II transcription bubble complex suggests the involvement of the non-template strand in facilitating polymerase translocation [48]. It raises a possible mechanism in which the formation of DNA secondary structure on the non-template strand could reduce/impair the participation of the non-template strand in the translocation process and consequently cause Pol II pausing. These mechanisms are clearly not mutually exclusive and possibly promote/enhance each other. It is likely that no single mechanism can account for pausing at every site. Further investigation of the mechanistic role of DNA secondary structure formation in Pol II pausing is needed.

The corresponding structures to those found on the non-template DNA strand could also be present on newly synthesized RNA due to sequence similarity. However, the significant portion of the RNA potentially capable of forming the corresponding structures would still be annealed to template DNA within the transcription complex. Therefore, it is unlikely that those structures could be formed on nascent RNA and impact Pol II pausing.

Many paused genes are known to have GC-rich promoter elements [49], and recent work has suggested a role for CpG islands located within human gene promoters in regulation of Pol II pausing at distinct sites [29]. The possible contribution of these GC-rich sequences in Pol II pausing as an energy barrier for the unwinding of duplex DNA by Pol II has been proposed [18]. However, we found that an enrichment of high GC content at the region of free energy minimum was just upstream of the pausing sites (Additional file 1: Figure S13A, B). If these same sequences were forming highly stable DNA–DNA bonds that acted as a barrier to Pol II elongation, thereby facilitating pausing, they would be downstream of the Pol II pausing sites. Moreover, regions with similar free energy minimums, but with either a low (< 48%, *n* = 200) or high (70 to 75%, n = 200) GC content in the 50-nt upstream region of pausing sites showed a similar pausing pattern (Additional file 1: Figure S15A, B). This indicates that although GC-rich sequence is associated with low DNA secondary structure free energy, nucleotide composition alone cannot explain Pol II pausing. Further, we found a strand bias around the TSS of paused genes (but not of non-paused genes), with the non-template strand having a significantly higher propensity to form the DNA secondary structures than the template strand (Additional file 1: Figure S3). Additionally, the *HSPA1B* mutant experimental data suggest that the free energy of secondary structure formation from the non-template strand is more strongly anti-correlated with Pol II pausing than the free energy of secondary structure formation from the template strand (Additional file 1: Figure S12B). These analyses suggest that the ability to form DNA secondary structures on the non-template strand could be a mechanism for how high GC content at TSSs influences Pol II pausing.

A study using a search algorithm [50] identified stem loop-containing quadruplex sequences on both template and non-template DNA in promoters and TSSs. In contrast, Eddy et al. [31] found the frequency of G4 quadruplex motifs peak at around 200 nt downstream of the TSS, and they displayed a non-template strand basis. One drawback of both studies is that there are no stability measurements to rank the structure-forming sequences, while in our study, each structure is associated with a free energy estimate that quantifies the structure formation potential.

The possibility that the mNET-seq data [37] that we used in our analysis could be influenced by Pol II backtracking is considered here. Backtracking occurs when RNA polymerase becomes disengaged from the 3′ end of nascent RNA and moves upstream. Backtracking can be relieved by moving forward to the previous positions or by TFIIS cleaving nascent RNA [51]. Studies have shown that backtracking depends on several factors, including the stability of the RNA:DNA hybrid, with the weaker the hybrid the more likely that backtracking will occur [52–54]. What is not clear is the degree of backtracking and the degree of TFIIS cleavage under the conditions in which the mNET-seq data were generated [37]. To search for possible traces of backtracking, for each pausing site located within a gene (we have defined the pausing sites as the strongest mNET-seq read spikes), we also identify the second most intense mNET-seq spikes and calculate the distance between the first and the second strongest mNET-seq spikes (*n* = 5263) (Additional file 1: Figure S16). The distribution of the distances shows that secondary spikes are within a few nucleotides of the strongest primary spikes. Therefore, if backtracking and TFIIS cleavage are present, we estimate that backtracking could at most change our result by ± 5 nt, which does not affect our overall results and conclusion.

Using a combination of Pol II pausing-related data sets, we developed a novel approach to identify pausing sites genome-wide. Our approach is entirely based on measurements rather than gene annotations as in previously proposed methods [22, 29, 31], and it provides the location of pausing sites at single nucleotide resolution. As a result, we can discover de novo pausing sites, and found nearly 6000 intergenic pausing sites in HeLa-S3 cells. Among them, some could be located at 3′ ends of annotated genes downstream of poly(A) signals, and some could be within genes that are not annotated in RefSeq. For example, 9% (*n* = 515) of those pausing sites are associated with long non-coding RNAs (lncRNAs) [55, 56], and recently, Pol II pausing has been shown to regulate transcription of a subset of lncRNAs in mammalian cells [57]. Further annotation of these sites will provide possible insights into the role of DNA secondary structure in Pol II pausing as well as the functional importance of specific non-coding RNAs.

In the in vitro pausing assay, *HSPA1B* mutant sequences showed a correlation of diminishing pausing signals associated with less stable DNA secondary structure by robust regression analysis, in which TGG and GGG mutants were effectively identified as outliers. These two mutants with more stable secondary structures than the WT sequence do not display stronger pausing in our assay. We speculate that sequence mutations could also influence sites of transcription factor binding. Analysis of transcription factor binding sites of all 16 *HSPA1B* sequences used in the assay with FIMO, a motif search tool, and HOCOMOCOv10 HUMAN mono transcription factors motifs database [58] shows that TGG and GGG mutants generate possible binding sites for TWST1 and MeCP2, respectively, suggesting that binding of these factors at these positions may influence the Pol II pausing process.

## Conclusions

We have presented an unbiased genome-wide analysis of Pol II pausing that led to the discovery of novel pausing sites located outside of annotated genes. Our study of DNA propensity to create secondary structures demonstrates a strong relationship of highly stable DNA secondary structure with Pol II pausing sites and their proximal location. Further, we present a mechanistic model that supports a role for DNA sequence features such as GC content and connects these features to potential recruitment of transcription factors in influencing Pol II pausing. Uncovering detailed mechanisms of how these DNA sequence elements and secondary structures establish and maintain paused polymerase awaits future studies.

## Methods

### Genome-wide DNA secondary structure predictions using Mfold and ViennaRNA

The human genomic DNA sequences for each individual chromosome (build GrCh37/hg19) were downloaded from the UCSC genome browser as FASTA files. DNA secondary structure prediction using Mfold [33] and ViennaRNA [34] was performed using a 300-nt sliding window with 150-nt step size across all chromosome sequences. For all genome-wide Mfold analyses, the default [Na^+^], [Mg^+^], and temperature inputs were 1.0 M, 0.0 M, and 37 °C, respectively. In vitro transcription conditions (60 mM KCl, 7 mM MgCl_2_, and 30 °C) were used for sequences examined using HeLa nuclear extracts. ViennaRNA analysis was performed using RNAfold v2.1.5 with thermodynamic parameters specifically for folding single-stranded DNA sequences (parameter file dna_mathews2004.par from the ViennaRNA package v2.1.5). G-quadruplex predictions were incorporated, and GU and lonely pairs were disallowed in the secondary structures. The free-energy (ΔG) value, in kcal/mol, of the most stable predicted secondary structure for each 300-nt window was used for the analyses. Undefined values that corresponded to sites of incomplete sequence in the human genome were not included in the analysis. The threshold used to determine sites of predicted highly stable DNA secondary structure was set as seven consecutive windows at which the predicted free energy value was in the lowest 5% of all values [40, 41].

### Functional genomics data analyses

If not stated otherwise, functional genomic data analyses and comparisons to gene annotations were performed using BEDTtools (v2.24.0) [59]. Heat maps of genomic data (ChIP-seq, GRO-seq, NET-seq, mNET-seq) were generated using NGS plot (v2.61). Free energy heat maps were visualized using Python (v3.5.2) with matplotlib.pyplot library (v2.0.2). If necessary, alignments of sequencing data were performed using bowtie2 (v2.1.0) and STAR (v2.5.1b) following descriptions from the original papers. Peaks were called with MACS2 (v2.0.9. 20111102) with default parameters. GRO-seq signal peaks were called for each strand separately. First reads were separated into those that aligned to opposite strands. Next, MACS2 was used to call peaks with the –no-model option. Finally, information about strand was added to the resulting lists of peaks, and top and bottom strand peak information was merged.

### Annotation of sites of predicted highly stable DNA secondary structures

RefSeq genes (build GRCh37/hg19) were downloaded from the UCSC Genome Browser and genomic regions were defined as follows:

- Promoter region ranging from − 1 knt to − 250 nt of the TSS
- TSS region ranging ± 250 nt from the TSS
- TTS region ranging ± 250 nt from the TTS
- Gene body region ranging from + 250 nt of TSS to − 250 nt of the TTS
- Intergenic region is the rest of the genome, not belonging to any of the four regions detailed above.

Free energy intervals (Mfold and ViennaRNA) that overlapped TSS regions were first assigned this annotation. Remaining regions were compared to TTS regions, promoter regions, and gene body regions. Free energy intervals that did not overlap these annotations were annotated as intergenic. The resulting number of intervals was normalized to the total size of each region.

### Association analysis of Mfold and ViennaRNA sites with RNA polymerase II features

Association analysis was performed using BEDTools software [59] to identify the number of Mfold or ViennaRNA sites that overlap with Pol II ChIP-seq peak intervals. Mfold or ViennaRNA sites were shuffled to generate random secondary structure sites consisting of the same number and size of the actual Mfold or ViennaRNA significant secondary structure sites. The ratio of the number of Mfold or ViennaRNA sites overlapping ChIP-seq peaks relative to the mean overlap for 10,000 shuffled sites is defined as a fold enrichment. The fold enrichment quantifies the association of the sites of predicted DNA secondary structure with Pol II binding compared to that expected by chance.

Quantitative read overlap analysis was performed by calculating Mfold or ViennaRNA sites coverage of Pol II ChIP-seq reads within Pol II ChIP-seq peaks. The Mfold or ViennaRNA sites were then shuffled 1000 times maintaining the same number and size of the actual sites of secondary structure. The ratio of the number of reads intersecting actual Mfold or ViennaRNA sites over the mean number of reads intersecting randomly shuffled sites was used as a fold enrichment measure of association between Mfold or ViennaRNA determined sites and Pol II ChIP-seq peak signal.

### Genomic regions selected for downstream analysis

To avoid bias towards genes with alternative splicing, we used the longest transcript for each gene in RefSeq (build GRCh37/hg19). Then, we filtered out any genes shorter than 660 nt, which is twice the length of the TSS-containing region (see TR definition) to avoid inaccurately classifying gene pausing status. Moreover, we discarded all genes with undetermined DNA sequences (i.e., nucleotides designated as “N”) within TSS ± 2 knt, and finally obtained 23,122 unique genomic loci.

### High resolution promoter-proximal DNA secondary structure free energy calculations

In order to model more accurately DNA secondary structure formation during transcription, ΔG calculations of secondary structure formation were performed on the non-template strand sequence of each gene region using a 30-nt window with a 1-nt step size. For the TSS ± 2 knt region of each gene, the most stable DNA secondary structure and its associated ΔG were determined for each 30-nt window using Mfold (v3.6) and ViennaRNA (v2.1.9) software with their default parameters for DNA calculations. Each ΔG value was assigned to the middle (15th nt) of the current window, resulting in a profile ranging from 1985 nt downstream to 1985 nt upstream of the TSS.

### Traveling ratio

The traveling ratio (TR) was calculated as previously described [22]. In brief, RefSeq genes (build GRCh37/hg19) were first stratified into two groups: intersecting and not intersecting with Pol II ChIP-seq peaks. Next, for Pol II-bound genes, we calculated coverage in two regions: − 30 to + 300 nt from the TSS and in the rest of the gene body. Next we determined the Pol II ChIP-seq read density by calculating the read coverage and dividing this by the length of the region. TR was calculated as a ratio of the density of reads in the − 30 to + 300 nt from the TSS region over the read density within the rest of the gene.

Based on the definitions above, all genes were divided into three groups: genes without Pol II binding, non-paused (TR ≤ 2), and paused (TR > 2) genes.

### Statistical analysis of mNET coverage

We first calculated the frequency of coverage within TSS-proximal regions. The low-read coverage, noise distribution was approximated by a power law function:

$$ {N}_x=0.965(5){x}^{-2.443(4)} $$

Its parameters were estimated using mNET-seq coverage ranging from 3 to 100. This normalized density was used as a null model distribution from which *p* values were calculated and FDR corrected. We applied a 5% FDR cutoff which corresponded to mNET read coverage > 4 reads.

### Generation of in vitro transcription templates and secondary structure mutants

Secondary structure mutants were generated with the Q5 Site-Directed Mutagenesis Kit (NEB). All primers used were designed using NEBaseChanger program and the pGEM-HSPA1B plasmid containing *HSPA1B* sequence (− 547 to + 293 relative to TSS). Generation and amplification of the mutated plasmid was carried out as described in the manufacturer’s protocol with the following PCR conditions: one cycle of 98 °C for 30 s; 25 cycles of 98 °C for 10 s, 65 °C for 10 s, 72 °C for 2 min; one cycle of 72 °C for 2 min. Annealing temperatures were adjusted depending on the primer being used to generate the mutants. Following the Kinase-Ligase-Dpn I reaction, mutated plasmids were transformed into SURE2 cells (Agilent) according to the manufacturer’s specifications with the exception of LB broth being used instead of SOC media. After transformation, colonies grown on LB + Ampicillin plates were selected and incubated for 16–18 h, and DNA was extracted using the GeneJET Plasmid MiniPrep Kit (Thermo Scientific). Mutations were validated with DNA sequencing and were verified to be the only change in the templates used in the pausing assay.

### In vitro transcription and pausing assay

All *HSPA1B* plasmids were subjected to restriction enzyme digestion with AccI and BsaAI to generate a 1290 bp DNA fragment containing *HSPA1B* sequence (− 547 to + 293 relative to TSS). DNA fragments for each mutant were gel-purified and biotinylated with a biotin conjugated forward primer (5′-biotin-GAACCATCACCCTAATCAAG-3′) using the PCR conditions: one cycle of 94 °C for 3 min; 25 cycles of 94 °C for 30 s, 62 °C for 30 s, and 72 °C for 30 s; one cycle of 72 °C for 7 min. DNA templates were cleaned with the PCR purification kit (BioBasic Inc.) and isolated with Dynabeads MyOne Streptavidin C1 (Invitrogen) beads. For in vitro transcription reactions, 125 ng of biotinylated fragments were immobilized on the beads at 2 fmol DNA per microgram of beads. DNA-bead complexes were washed with double bead volume of 1X B&W buffer. DNA–bead complexes were then washed with double bead volume of transcription buffer (13 mM HEPES pH 7.6, 60 mM KCl, 0.1 mM EDTA, 7 mM DTT, 13% glycerol, 7 mM MgCl_2_, 10 μM ZnCl_2_, 10 mM creatine phosphate). DNA–bead complexes were incubated for 30 min at room temperature with nuclear extract prepared following the Cold Spring Harbor HeLa Nuclear Extract protocol [60] using HeLa cells (Texcell, Inc.). DNA–protein complexes were pulled down and loosely bound proteins were washed away with ten bead volumes of TW buffer (13 mM HEPES pH 7.6, 60 mM KCl, 100 μM EDTA, 7 mM DTT, 13% glycerol, 7 mM MgCl_2_, 10 μM ZnCl_2_, 0.0125% NP-40). DNA–protein complexes were resuspended in 23 μL of transcription buffer, and 1 μL of an rNTPs mixture (0.4 mM rCTP, 10 mM rATP, 10 mM rUTP, 10 mM rGTP) and 1 μL of [α-P^32^] rCTP (Perkin Elmer) were added to the solution. The transcription reaction was allowed to progress for 30 min at 30 °C. The reaction was stopped with 175 μL of stop solution (0.3 M Tris-HCl pH 7.4, 0.3 M sodium acetate, 0.5% SDS, 2 mM EDTA, 3 μg/ml tRNA). RNA transcripts were cleaned by phenol/chloroform/isoamyl alcohol (25:24:1) extraction and ethanol precipitation. RNA transcripts were run on a 6% polyacrylamide/7 M urea denaturating gel, and the images were developed with phosphorimaging.

### Structural probing analysis with mung bean nuclease

Oligonucleotides were designed for WT and two mutants (CGG and CGA) from the + 46- to the + 75-nt position of the *HSPA1B* non-template strand sequence, with the respective point mutations at the + 64-nt position. Each oligo was labeled on the 5′ end with T4 DNA kinase and [γ-P^32^] ATP. Labeled DNAs (16 ng) were first incubated in modified transcription buffer (13 mM HEPES pH 7.6, 60 mM KCl, 0.1 mM EDTA, 1 mM DTT, 7 mM MgCl_2_, 10 μM ZnCl_2_) for 30 min at 30 °C to allow the DNA secondary structure formation, and then 2 U of mung bean nuclease were added to a final reaction of 20 μL. The reactions were allowed to proceed for 40 min at 30 °C, stopped with equal volume of formamide loading buffer and boiled for 5 min, and analyzed on an 8% polyacrylamide/7 M urea denaturating gel.

### Data availability

High-throughput sequencing data used in this study have been downloaded from Gene Expression Omnibus (GSE numbers), Sequence Reads Archive (SRA), or from ENCODE project [61] through UCSC Genome Browser (GSE and wgEncode numbers) [62]: for Hela-S3 cells, Pol II ChIP-seq (GSM935395, wgEncodeEH000613) [61], Pol II ChIP-seq peaks files (GSM935395, wgEncodeEH000613) [61], GRO-seq (GSE62047) [35], NET-seq (GSE61332) [36], and mNET-seq (GSE60358) [37]; for A549 cells, Pol II ChIP-seq (GSM822288, wgEncodeEH002079) [61], Pol II ChIP-seq peaks files (GSM822288, wgEncodeEH002079) [61]; for GM12878 cells, Pol II ChIP-seq (GSM935412, wgEncodeEH000626) [61], Pol II ChIP-seq peaks files (GSM803355, wgEncodeEH001463) [61]; for H1-hESC cells, Pol II ChIP-seq (GSM822300, wgEncodeEH000563) [61], Pol II ChIP-seq peaks files (GSM803366, wgEncodeEH001499) [61]; for K562 cells, Pol II ChIP-seq (GSM935358, wgEncodeEH000616) [61], Pol II ChIP-seq peaks files (GSM803410, wgEncodeEH001633) [61]; for HCT116 cells, Pol II ChIP-seq (GSE60106) [63]; for human Ntera 2 cells, DRIPc-seq (GSE70189) [47]; for Raji cells, Pol II ChIP-seq (GSM935461, wgEncodeEH001761) [61] and ssDNA-seq (SRA072844) [39]; for NHEK cells, Pol II ChIP-seq (GSE30226) [61] and G4 ChIP-seq (GSE76688) [38].

## Additional files

Additional file 1: Supplemental figures, tables, methods, and references. **Figures S1–S16** and **Tables S1–S5**. (PDF 6024 kb)

Additional file 2: A list of 7972 pausing sites located within RefSeq gene bodies. (DAT 368 kb)

Additional file 3: A list of 5959 pausing sites located outside of RefSeq gene bodies. (DAT 234 kb)
