# Supplementary material for: Alternative DNA secondary structure formation affects RNA polymerase II promoter-proximal pausing in human
Source: Genome Biol. 2018 Jul 12;19:89. doi: 10.1186/s13059-018-1463-8 (PMC6042338; doi:10.1186/s13059-018-1463-8)
Supplement: Supplementary file 1 — Supplemental figures, tables, methods, and references. Figures S1–S16 and Tables S1–S5. (PDF 6024 kb) [file 13059_2018_1463_MOESM1_ESM.pdf]

Additional File 1:

Table S1. Genome-wide DNA secondary structure prediction statistics.

Table S2. Gene location of highly stable DNA secondary structure.

Table S3. Number of genes classified as paused, non-paused, and no Pol II based on traveling ratio.

Table S4. DNA secondary structure folding free energy is significantly lower at the TSS of pause genes.

Table S5. Perturbation of secondary structure free energy correlates with Pol II pausing.

Figure S1. Significant enrichment of Pol II ChIP-seq reads at sites of highly stable secondary structures.

Figure S2. The promoter-proximal regions of paused genes have significantly lower ΔG compared to non-paused genes or genes without Pol II binding.

Figure S3. Differences in propensity to form DNA secondary structure between non-template and template strand.

Figure S4. GRO-seq and NET-seq signals at the TSS of paused genes.

Figure S5. Highly stable DNA secondary structures predicted by the Mfold or the ViennaRNA are located upstream of the paused sites as demonstrated by single-nucleotide resolution signals of mNET-seq.

Figure S6. Highly stable DNA secondary structures are preferentially located up to 50 nt upstream of the mNET-seq spike.

Figure S7. Definition of pausing site based on Pol II ChIP-seq, GRO-seq, NET-seq, and mNET-seq signals.

Figure S8. Highly stable DNA secondary structures preferentially form just upstream from pausing sites.

Figure S9. Heat maps showing a strong correlation among the secondary structure footprints, the predicted low free energies, and the location of Pol II.

Figure S10. Gene examples showing the positions of the secondary structure footprints, the low free energies and Pol II at the TSS ± 2kb region.

Figure S11. Genes' pausing status is highly correlated among eight cell lines.

Figure S12. *HSPA1B* mutations.

Figure S13. Genomic features at Pol II pausing sites.

Figure S14. R-loops at pausing sites.

Figure S15. GC content and Pol II pausing.

Figure S16. Trace of backtracked polymerase.

Supplemental Methods and References

Table S1 Genome-wide DNA secondary structure prediction statistics.

	Mfold (kcal/mol)	ViennaRNA (kcal/mol)
Average	-26.8	-30.2
SD	9.5	11.9
Minimum	-180.5	-937.4
Maximum	8.1	0
95th percentile value	-43.6	-51.7
Highly stable DNA secondary structure sites*	15,826	14,778
* sites of highly stable DNA secondary structures defined as having at least seven consecutive segments with a free energy value in the top 5% most stable structures predicted across the genome		

Table S2 Gene location of highly stable DNA secondary structure.

MFold site locations:						
	Total	at promoter	at TSS	within gene body	at TTS	within intergenic region
Number of sites	15826	51	4419	6823	500	4033
Fraction of sites [%]	100	0.3	27.9	43.1	3.2	25.5
Normalized fraction of sites [per 1kb]	5.86*	0.18	23.03	0.32	2.61	0.19
Vienna site locations:						
	Total	at promoter	at TSS	within gene body	at TTS	within intergenic region
Number of sites	14778	49	4326	6342	457	3604
Fraction of sites [%]	100	0.3	29.3	42.9	3.1	24.4
Normalized fraction of sites [per 1kb]	5.47*	0.18	24.14	0.32	2.55	0.18

*Average site density genome-wide

Table S3 Number of genes classified as paused, non-paused, and no Pol II based on traveling ratio.

Cell line	paused genes	non-paused genes	no Pol II genes	Total	% of paused gene of total Pol II-bound genes
A549	8040	1803	13279	23122	82
GM12878	9178	1729	12215	23122	84
HCT116	11743	1356	10023	23122	90
HeLa-S3	11038	1787	10297	23122	86
H1-hESC	10185	1938	10999	23122	84
K562	8811	2154	12157	23122	80
NHEK	9072	2272	11778	23122	80
Raji	10874	2093	19155	23122	84

Table S4 DNA secondary structure folding free energy is significantly lower at the TSS of pause genes.

Cell type	Resolution of free energy calculation*	Software	Mean free energy within TSS +/- 250 nt			T-test P-values	
			no Pol II	non-paused	paused	non-paused < paused	no Pol II < paused
HeLa	30	MFold	-2.20	-2.41	-3.14	$< 2.2x10^{-16}$	$< 2.2x10^{-16}$
		Vienna	-2.42	-2.71	-3.65	$< 2.2x10^{-16}$	$< 2.2x10^{-16}$
	300	MFold	-38.64	-42.09	-51.11	$< 2.2x10^{-16}$	$< 2.2x10^{-16}$
		Vienna	-46.06	-50.35	-61.97	$< 2.2x10^{-16}$	$< 2.2x10^{-16}$
hESC	30	MFold	-2.40	-2.16	-3.05	$< 2.2x10^{-16}$	$< 2.2x10^{-16}$
		Vienna	-2.69	-2.39	-3.51	$< 2.2x10^{-16}$	$< 2.2x10^{-16}$
	300	MFold	-41.43	-38.73	-50.20	$< 2.2x10^{-16}$	$< 2.2x10^{-16}$
		Vienna	-49.65	-45.90	-60.66	$< 2.2x10^{-16}$	$< 2.2x10^{-16}$
A549	30	MFold	-2.51	-2.14	-3.04	$< 2.2x10^{-16}$	$< 2.2x10^{-16}$
		Vienna	-5.33	-4.54	-6.31	$< 2.2x10^{-16}$	$< 2.2x10^{-16}$
GM12878	30	MFold	-2.37	-2.37	-3.14	$< 2.2x10^{-16}$	$< 2.2x10^{-16}$
		Vienna	-5.03	-5.05	-6.48	$< 2.2x10^{-16}$	$< 2.2x10^{-16}$
K562	30	MFold	-2.35	-2.52	-3.14	$< 2.2x10^{-16}$	$< 2.2x10^{-16}$
		Vienna	-5.00	-5.34	-6.51	$< 2.2x10^{-16}$	$< 2.2x10^{-16}$
HCT116	30	MFold	-2.11	-2.31	-3.18	$< 2.2x10^{-16}$	$< 2.2x10^{-16}$
		Vienna	-4.54	-4.99	-6.60	$< 2.2x10^{-16}$	$< 2.2x10^{-16}$

* The free energy of secondary structure formation was calculated by Mfold and ViennaRNA for the non-template strand of each gene with either a 30-nt or 300-nt sliding window and a 1-nt step size.

Table S5. Perturbation of secondary structure free energy correlates with Pol II pausing.

Mutant	Fraction of paused transcripts	Cumulative ΔG difference (kcal/mol)*
TGT	0.61	10.16
AGG	0.69	-8.46
TGG	0.70	-12.07
CGT	0.72	7.78
CGA	0.73	10.55
TGC	0.77	-0.58
AGT	0.78	12.06
GGT	0.79	8.64
TGA	0.81	10.55
GGG	0.82	-12.63
GGC	0.87	-1.26
GGA	0.89	-5.00
AGC	0.89	-2.79
AGA	0.91	10.55
CGC (WT)	0.95	0.00
CGG	0.96	-5.17

* Each variant could potentially affect the secondary structure formation in 61 segments (30 nt windows with a 1 nt step): the first segment is nt 33-62 due to the mutation at nt 62, and the last segment is nt 64-93 due to the mutation at nt 64. Therefore, to account for all these possible secondary structures and their associated free energies, the cumulative free energy difference between the variant and the wild type is shown for each variant.

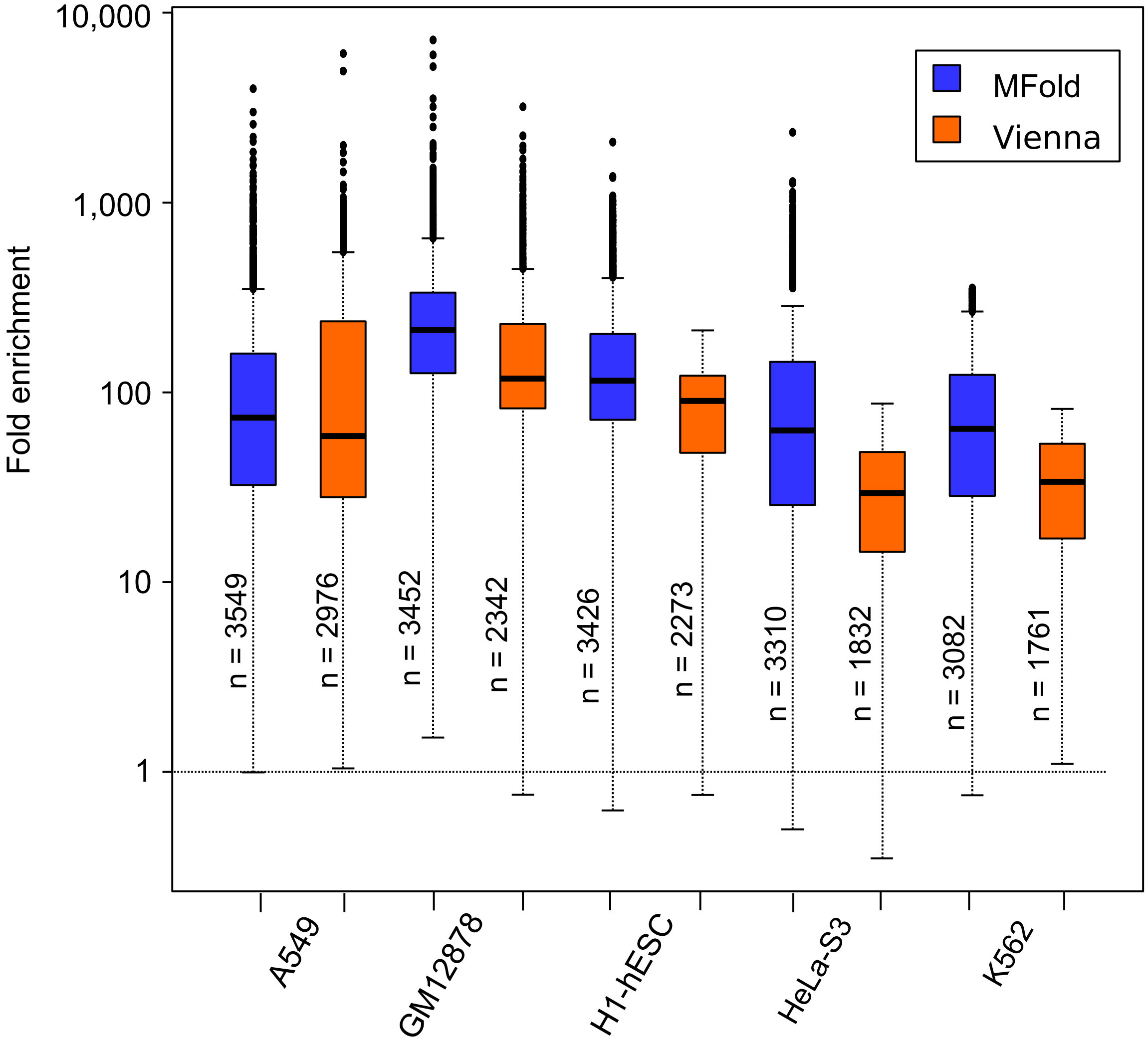

Figure S1. Significant enrichment of Pol II ChIP-seq reads at sites of highly stable secondary structures. Fold enrichments in Pol II ChIP-seq read coverage for Mfold and Vienna sites over a mean coverage of randomly shuffled sites are significant in all five cell lines ($p < 0.002$, permutation analysis). Dotted line at fold enrichment = 1 denotes no change.

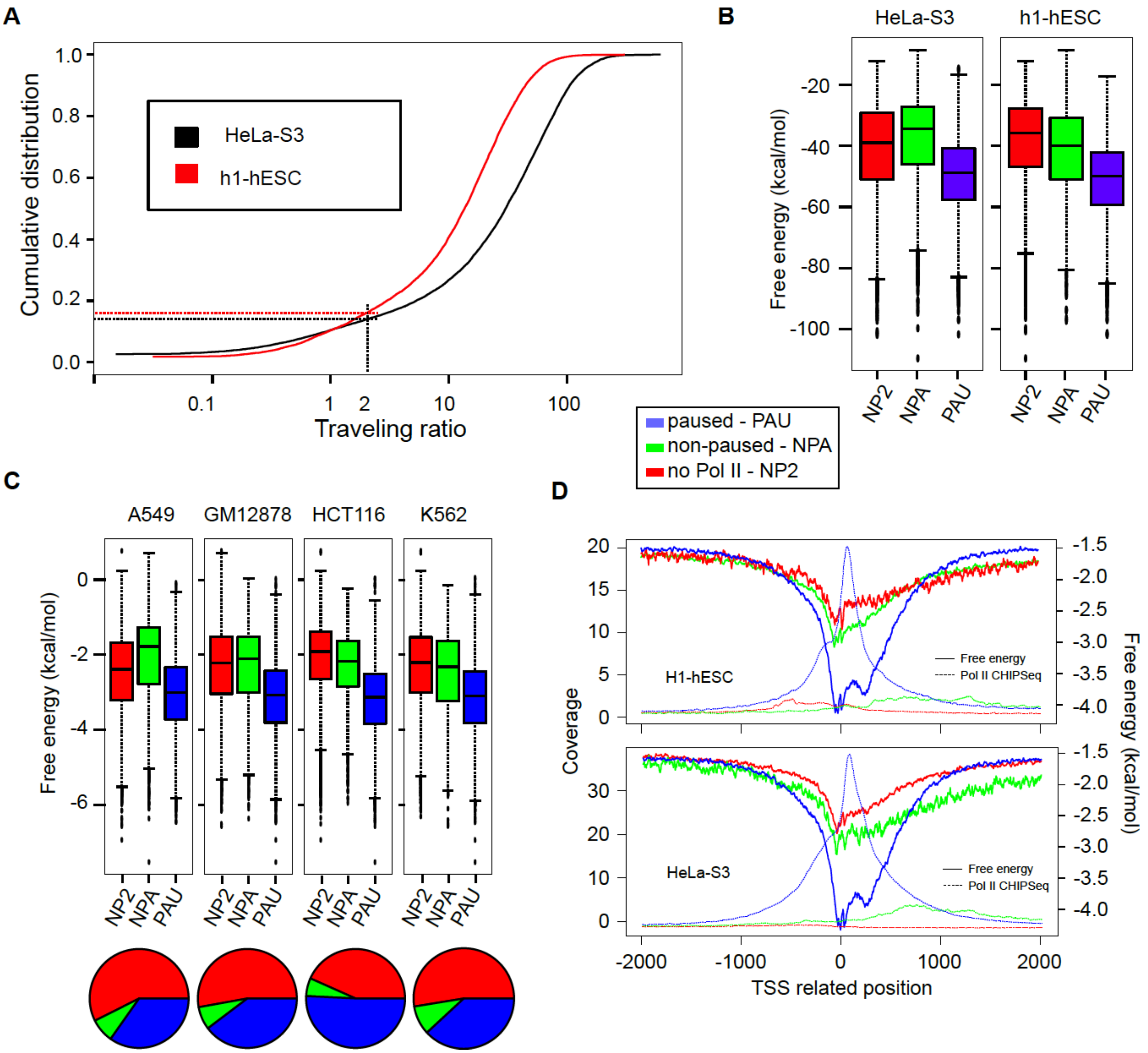

Figure S2. The promoter-proximal regions of paused genes have significantly lower Δ G compared to non-paused genes or genes without Pol II binding. A. Cumulative distributions of traveling ratio in HeLa-S3 (black) and H1-hESC (red) cell lines are shown. **B.** Mean free energy at the TSS ± 250nt region of genes in HeLa-S3 (left) and H1-hESC (right) cell lines. Free energy was calculated within a 300-nt sliding window with a 150-nt step size. Genes were grouped based on their traveling ratio into three groups: no Pol II (NP2, red), non-paused (NPA, green), and paused (PAU, blue). **C.** Mean free energy at the TSS ± 250 nt region of genes in four cell lines: A549, GM12878, HCT116, and K562. Free energy was calculated with a 1-nt step size within a 30-nt sliding window. Genes were grouped as in **B.** $*p < 2.2x10^{-16}$, t-test (Supplemental Table S4). **D**. Average free energy profiles (solid line) and average Pol II ChIP-seq coverage profiles (dotted line) are shown in two cell lines: H1-hESC (top) and HeLa-S3 (bottom). Paused, non-paused, and no Pol II genes are shown in blue, green, and red, respectively. The Mfold analysis was used for **A-C** and the ViennaRNA program was used for **D**.

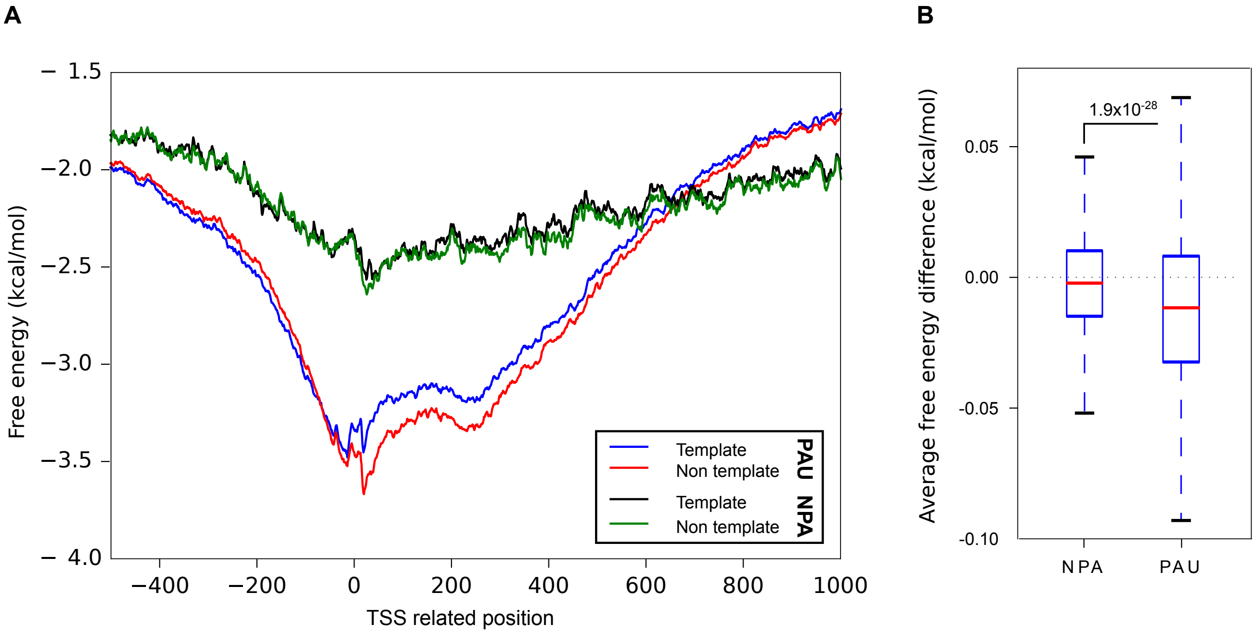

Figure S3. Differences in propensity to form DNA secondary structure between non-template and template strand. A. Average free energy profiles are shown for paused (PAU) and non-paused (NPA) genes with non-template or template strand sequences at TSS -500 nt to TSS +1000 nt. **B.** Box plot shows differences in free energies between non-template and template strands for paused (PAU) and non-paused genes (NPA). Paused genes have significantly (t-test) higher propensity to form secondary structure on the non-template strand, while this is not observed for non-paused genes.

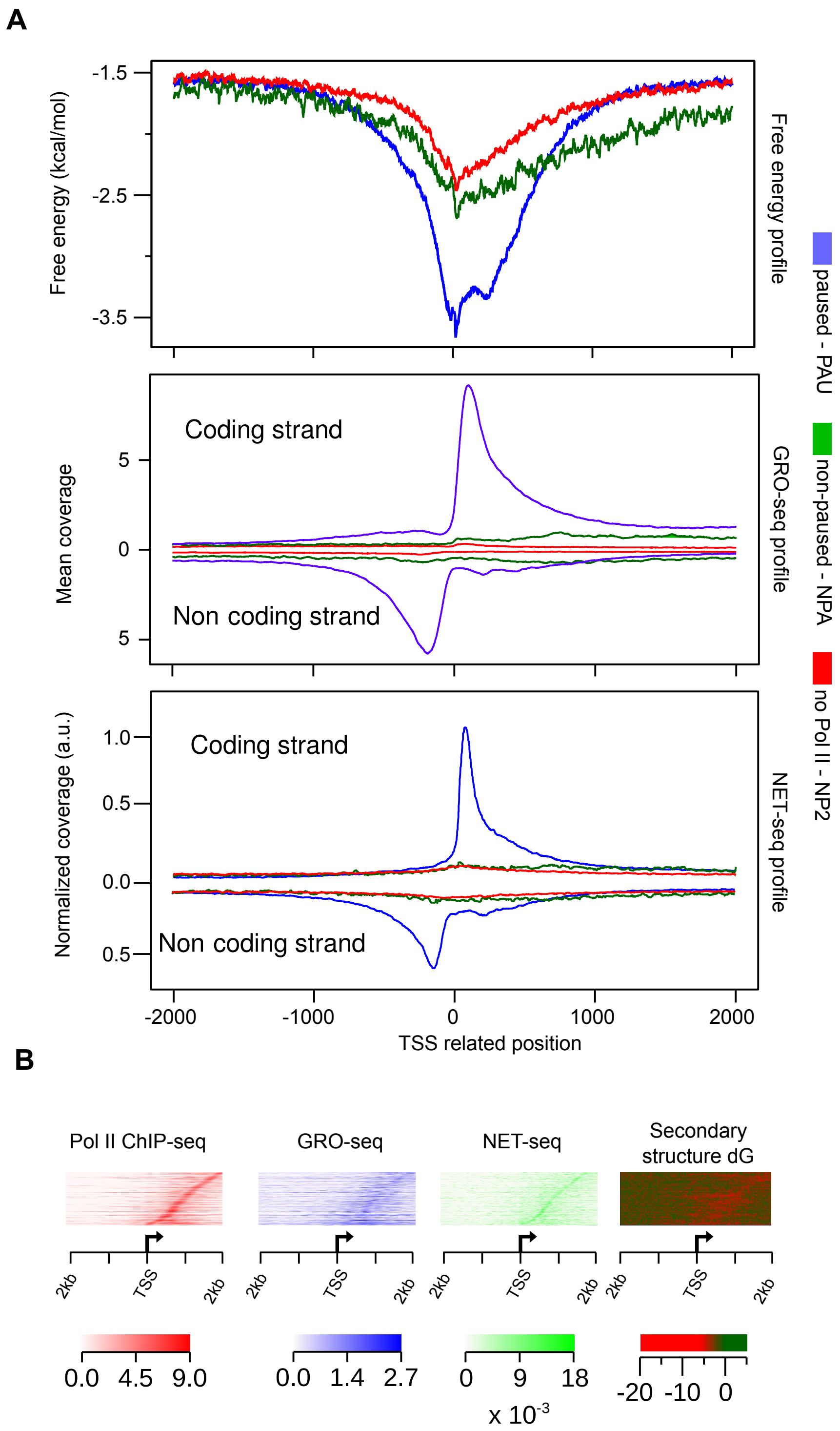

Figure S4. GRO-seq and NET-seq signals at the TSS of paused genes. A. The average profile of secondary structure free energy (top) and the average coverage profile of GRO-seq (middle) and NET-seq (bottom) at the TSS ± 2 knt region in HeLa-S3 cells are shown. Coverages were calculated for coding and non-coding strands separately. Genes are grouped by traveling ratio in three groups: no Pol II (red), non-paused (green), and paused (blue) **B.** Heat map representations of Pol II ChIP-seq (red), GRO-seq (blue), NET-seq (green) coverage, and free energy (green-red) profiles in HeLa-S3 cells are shown for the TSS ± 2 knt region of non-paused genes (n = 655). Non-paused genes were ordered according to the distance of each Pol II ChIP-seq peak summit from the TSS.

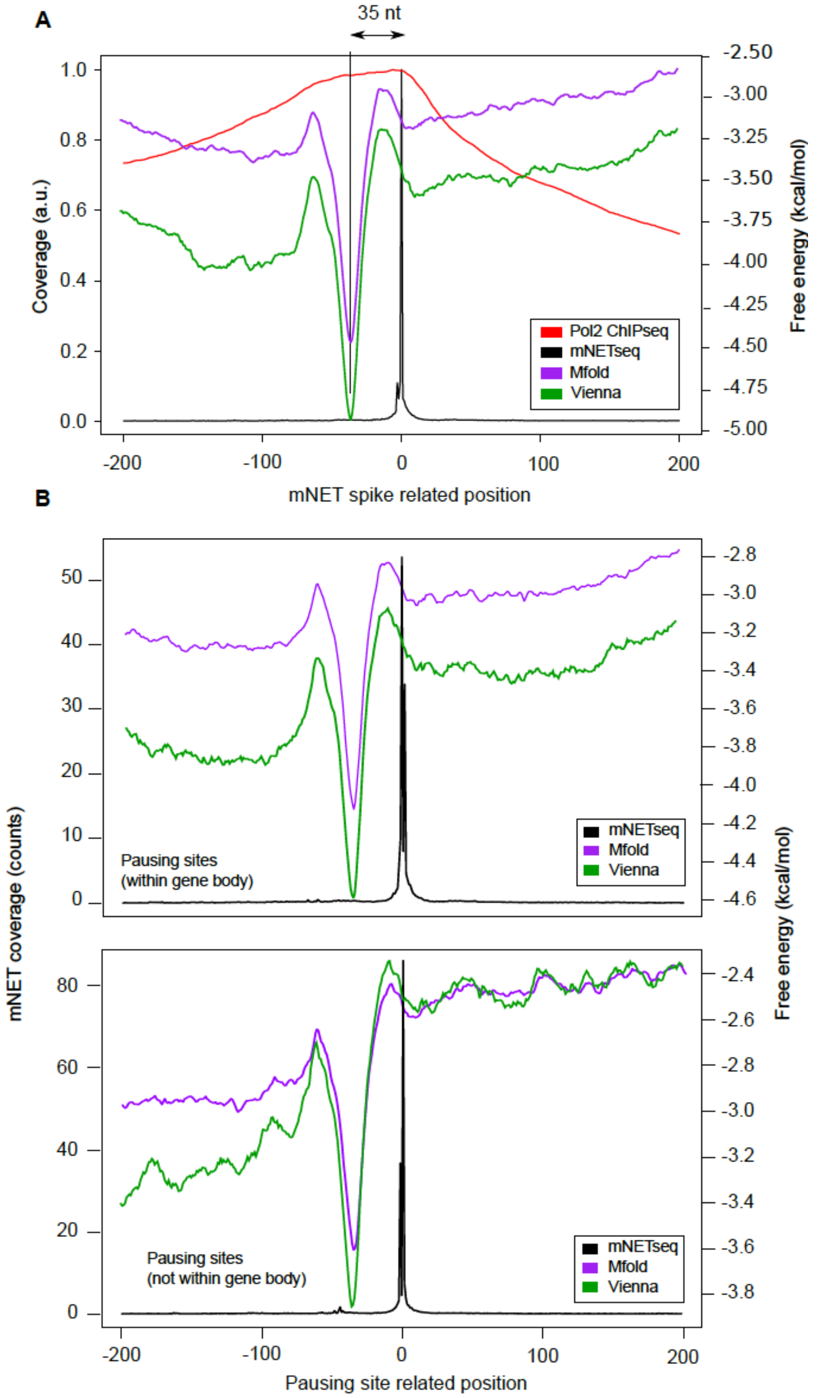

Figure S5. Highly stable DNA secondary structures predicted by the Mfold or the ViennaRNA are located upstream of the paused sites as demonstrated by single-nucleotide resolution signals of mNET-seq. **A.** An average free energy profile (purple-Mfold; green-ViennaRNA) and average Pol II coverage (red) centered at the strongest mNET-seq read spike (black) are shown (n=10428). At the average free energy of -3.25 and -3.7 kcal/mol for the Mfold and ViennaRNA, respectively, DNA secondary structures are, on average, about 10 nt to 40 nt upstream from the peak of the highest mNET-seq read spikes. **B.** Average profile plot of mNET-seq coverage (black) and secondary structure free energy (purple-Mfold; green-ViennaRNA) at Pol II pausing sites are shown for pausing sites located within human RefSeq annotated genes (top panel, n=7972) and pausing sites located outside of human annotated genes (bottom panel, n=5959). Inclusion of G-quadruplexes did not change the shape or position of the sharp free energy minimum relative to the mNET-seq peaks, but it did lower the ΔG of the overall average curve.

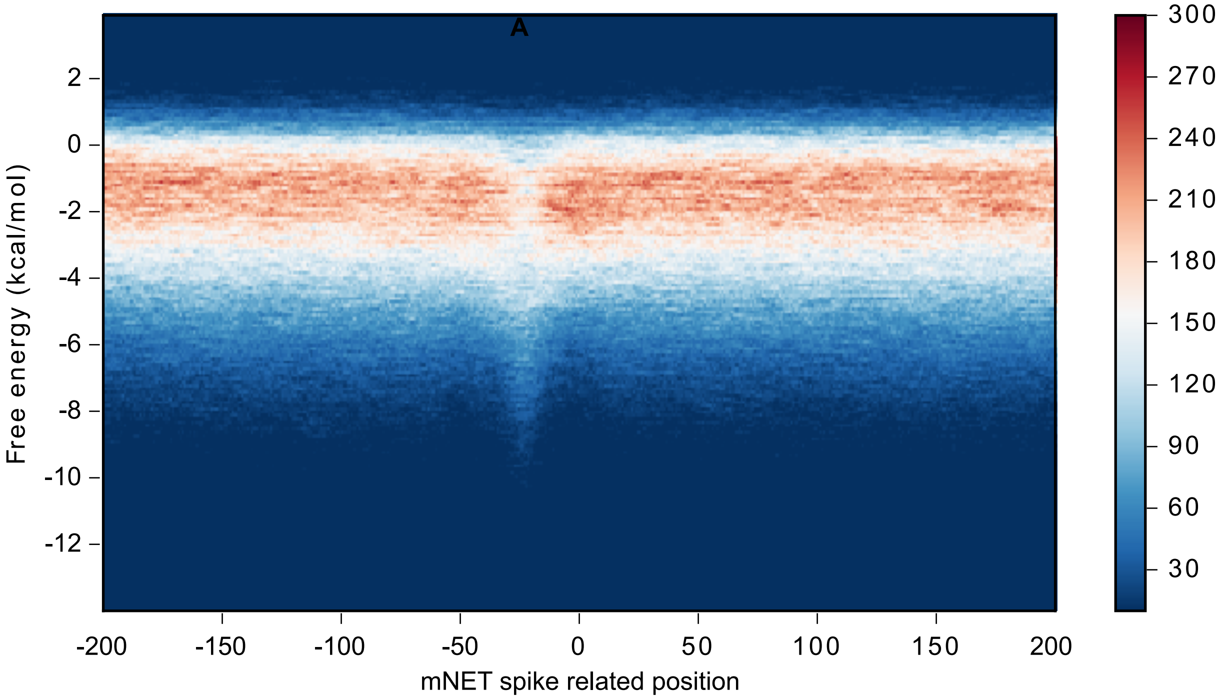

Figure S6. Highly stable DNA secondary structures are preferentially located up to 50 nt upstream of the mNET-seq spike. The two-dimensional frequency plot of secondary structure free energy of the non-template strand around TSS proximal mNET-seq spikes is shown (n = 10428). Color represents abundance of free energies at each nucleotide (red corresponds to more frequent values of free energy). The average plot of these data is shown in Fig. 4B.

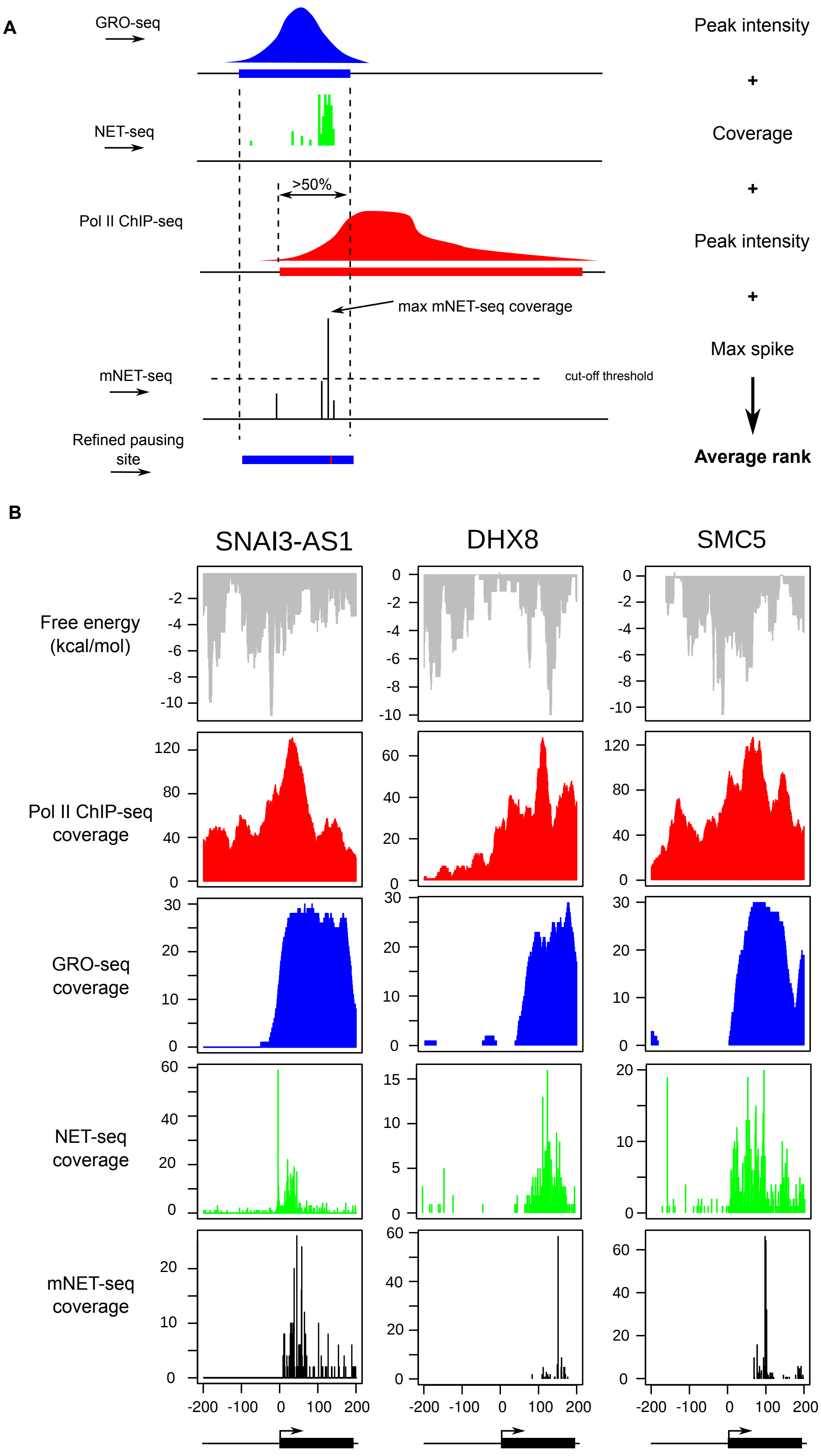

A
GRO-seq
NET-seq
>50%
Pol II ChIP-seq
max mNET-seq coverage
mNET-seq
cut-off threshold
Refined pausing site
Peak intensity
+
Coverage
+
Peak intensity
+
Max spike
Average rank
B
SNAI3-AS1
DHX8
SMC5
Free energy (kcal/mol)
Pol II ChIP-seq coverage
GRO-seq coverage
NET-seq coverage
mNET-seq coverage
-200 -100 0 100 200

Figure S7. Definition of pausing site based on Pol II ChIP-seq, GRO-seq, NET-seq, and mNET-seq signals. A. Schematic representation of the criteria used to define pausing sites: GRO-seq peak (blue) is required to intersect significantly (>50%) with Pol II peak (red). Then, based on the average of the ranks of each signal intensity (i.e., Pol II ChIP-seq, GRO-seq, NET-seq, and mNET-seq), pausing loci are ranked from 1 (the strongest) to 13931 (the weakest). Pausing sites are determined to be at the highest mNET spikes. **B.** Free energy (grey), Pol II ChIP-seq (red), GRO-seq (blue), NET-seq (green), and mNET-seq (black) profiles are shown at the TSS ± 200 nt of three genes, *SNAI3-AS1*, *DHX8*, and *SMC5* whose pausing ranks are 7, 239 and 486, respectively.

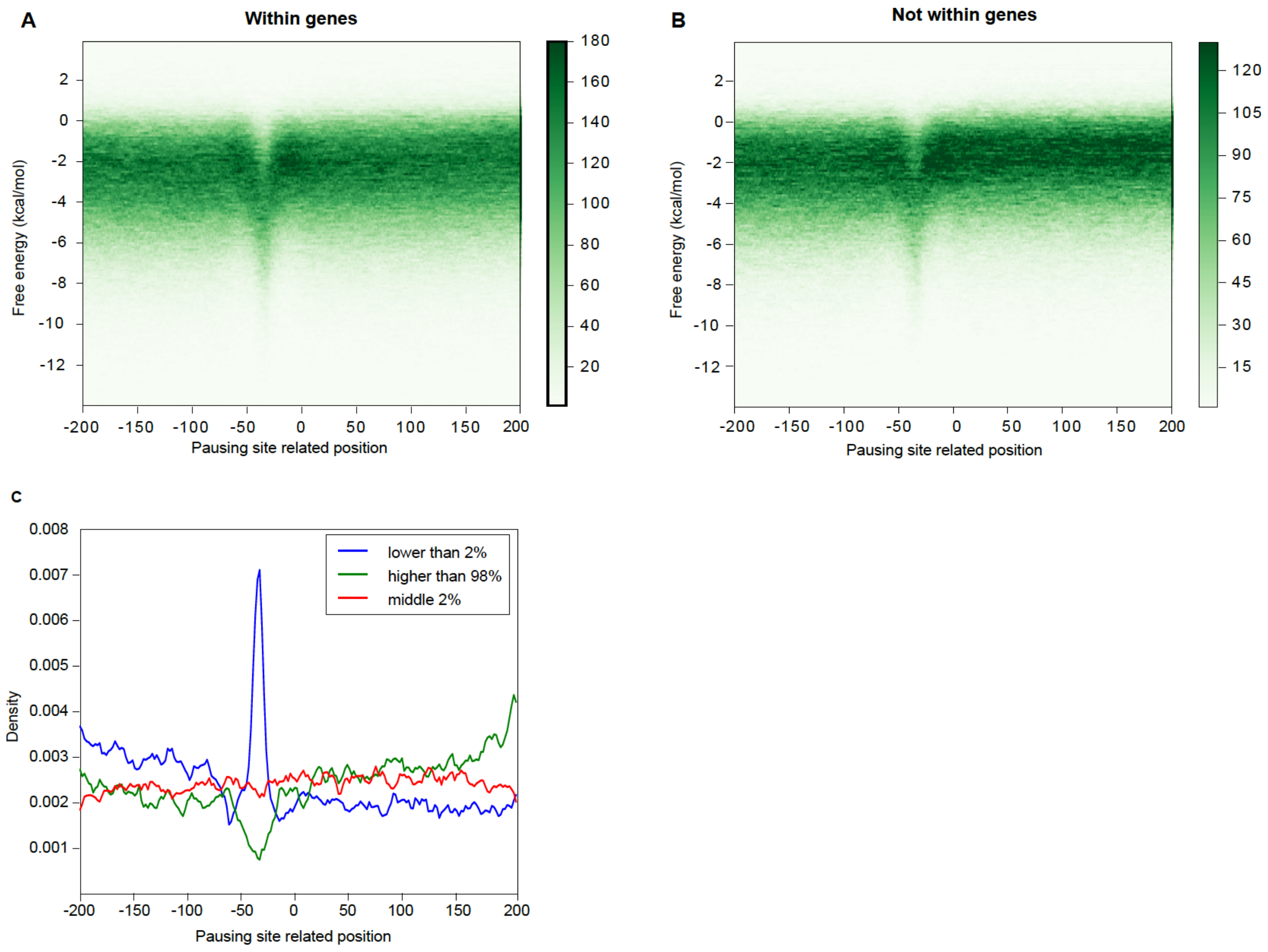

Figure S8. Highly stable DNA secondary structures preferentially form just upstream from pausing sites. Frequency plots of free energies for pausing sites located within genes (n=7972) (**A**) and outside of the genes (n=5959) (**B**) are presented. Color intensity represents abundance of DNA secondary structures of given free energy at each nucleotide at pausing sites ± 200nt. The "0" pausing site-related position on the x-axis is the nucleotide where the highest mNET-seq read spike is in each pausing site. The data are used to generate the average profiles in Fig. 4D. **C.** Density of the locations of most stable (2%), average (2%), and less stable (2%) of total secondary structures around pausing sites ± 200 nt for each pausing site. The "0" pausing site-related position on the x-axis is the nucleotide where the highest mNET-seq read spike is in each pausing site.

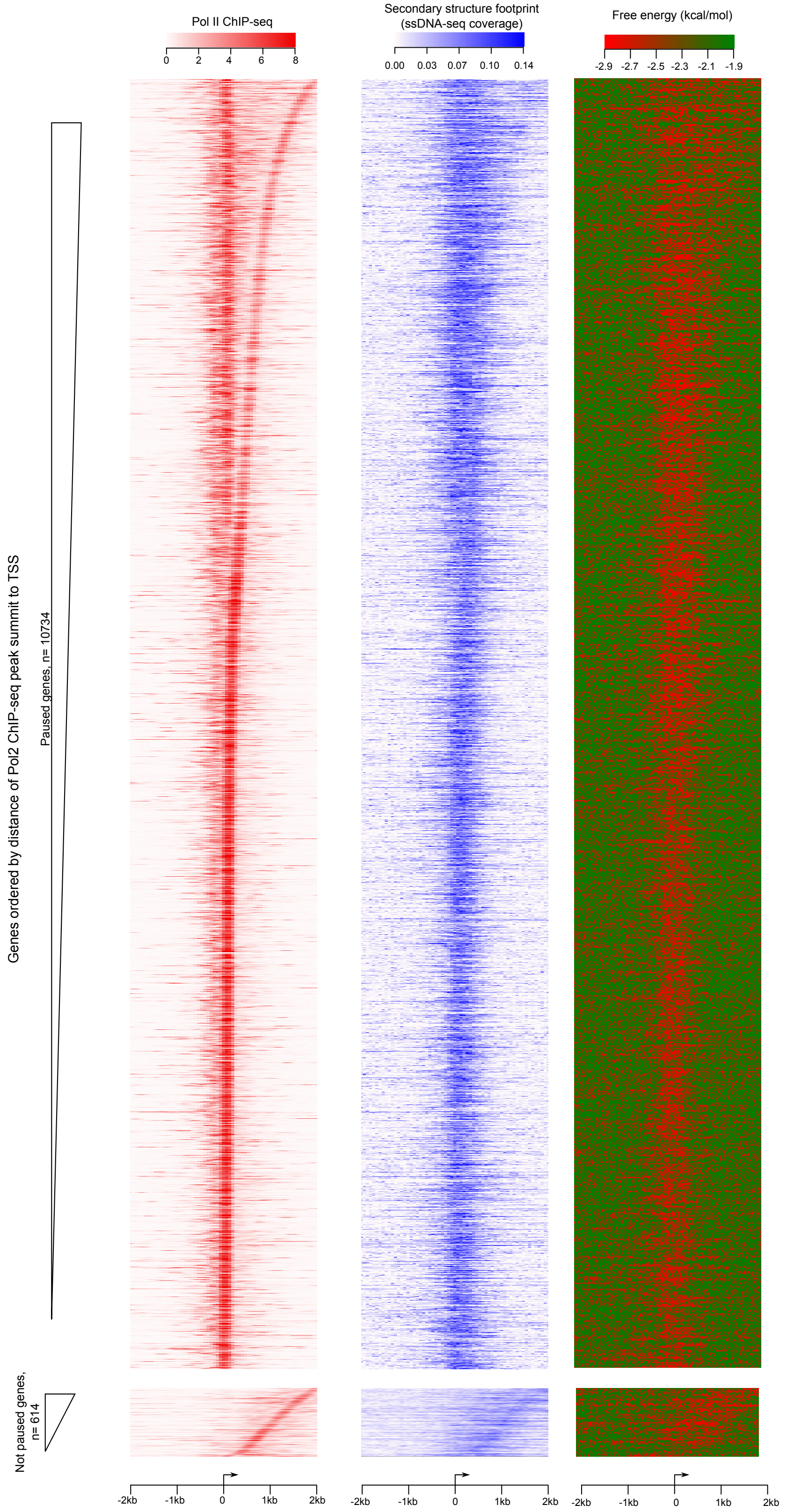

Figure S9. Heat map representations of Pol II ChIP-seq (red), and secondary structure-probing coverage (blue), and predicted free energy (green-red) profiles in Raji cells are shown at the TSS ± 2 knt region. Paused (n = 10734) and non-paused (n= 614) genes were ordered by the distance of the Pol II ChIP-seq peak summit from each gene's TSS. The data are used to generate the average profiles in Fig. 6A.

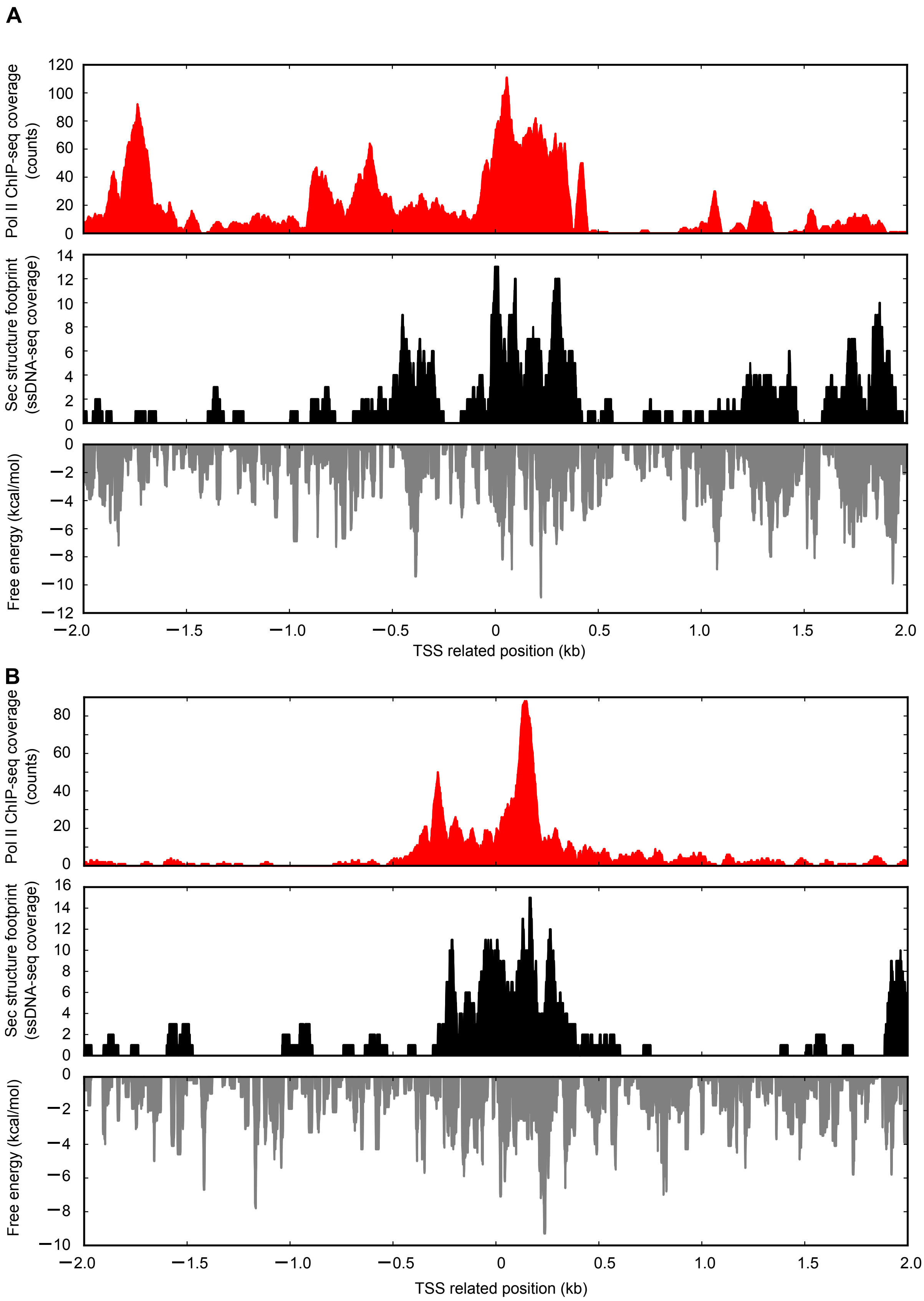

Figure S10. Pol II ChIP-seq (top), *in vivo* secondary structure footprints (middle) and predicted free energy (bottom) profiles at TSS ± 2kb of the *Myc* (**A**) and the *CDK4* (**B**) genes.

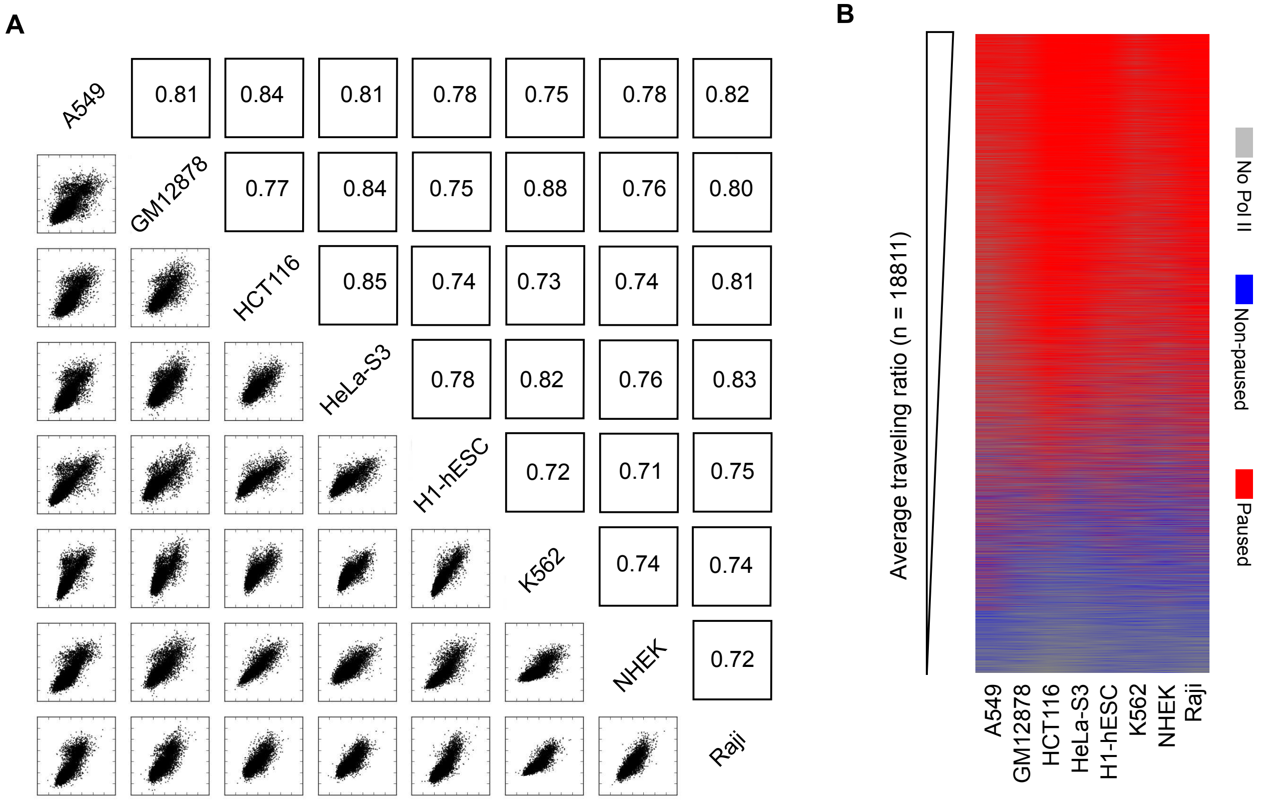

C

Y cell line \ X cell line		# of genes (paused in X but non-paused in Y)							
		**A549**	**GM12878**	**HCT116**	**HeLa-S3**	**H1-hESC**	**K562**	**NHEK**	**Raji**
# of genes (paused in Y but non-paused in X)	**A549**	----	252	201	397	448	432	650	487
	**GM12878**	262	----	234	360	369	413	564	453
	**HCT116**	260	389	----	384	359	568	562	579
	**HeLa-S3**	199	277	145	----	325	405	469	436
	**H1-hESC**	274	246	200	384	----	394	554	470
	**K562**	224	214	181	264	295	----	461	427
	**NHEK**	259	257	182	352	321	395	----	431
	**Raji**	275	279	211	350	386	494	559	----

Figure S11. Genes' pausing status is highly correlated among eight cell lines. A. Matrix presents correlations of traveling ratio among eight human cell lines: A549, GM12878, HCT116, HeLa-S3, H1-hESC, K562, NHEK, and Raji. Upper-right corner shows Spearman correlation coefficients. Lower-left corner shows scatter plots (black dots) of traveling ratio among cell lines. **B.** The heat map displays genes' pausing status (no Pol II – gray, non-paused – blue, or paused – red) across six cell lines. Genes were ordered by average traveling ratio. **C.** The number of genes with switched pausing status in different cell lines are presented.

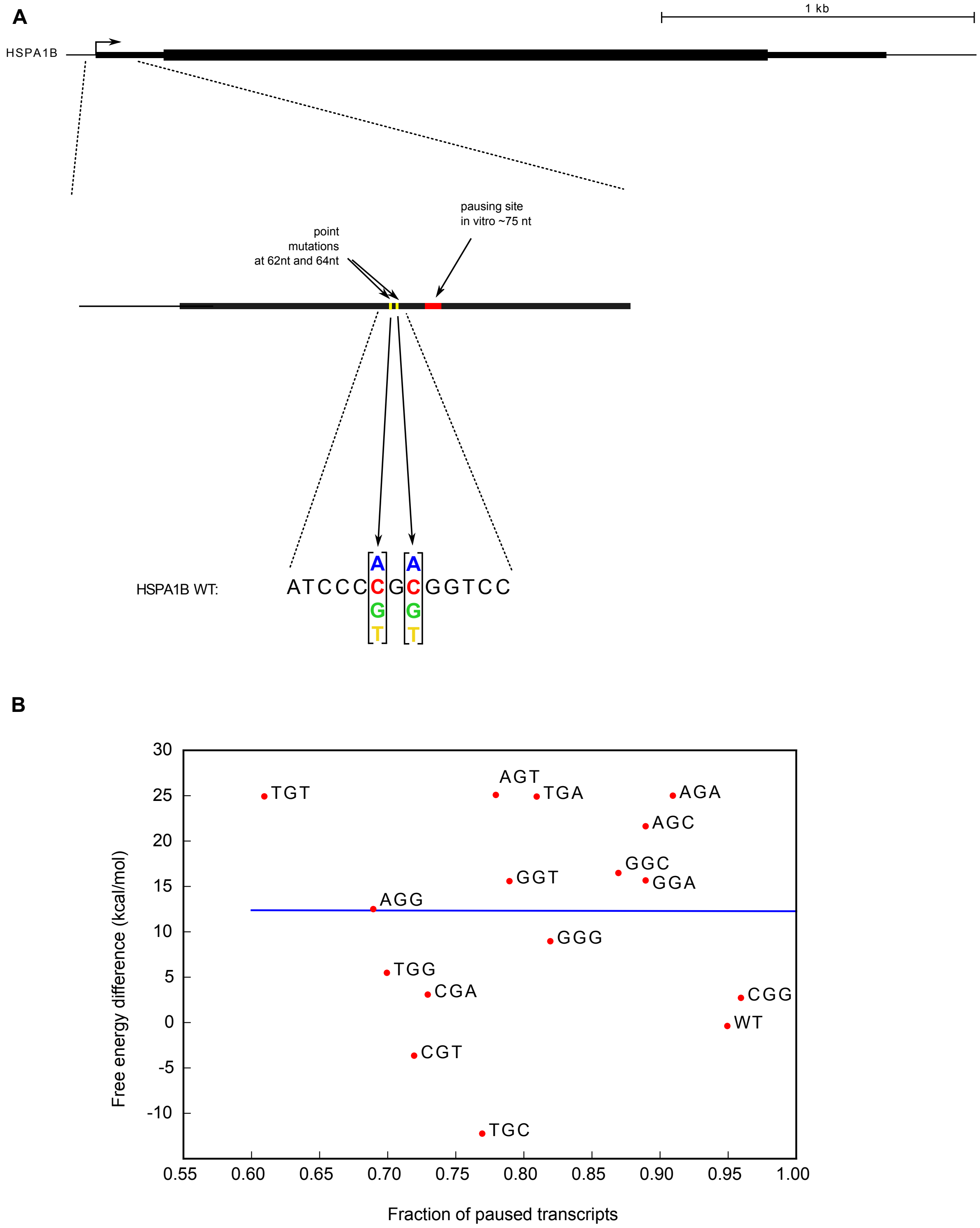

Figure S12. ***HSPA1B*** **mutations. A.** Schematic representation of mutations in the *HSPA1B* gene is shown. Point mutations at nt 62 and 64 downstream from the TSS and just upstream from the *in vitro* pausing sites (centered around nt 75 from the TSS) generate sixteen mutants. **B**. The dependence of cumulative free energy (on template strand) difference and experimentally measured fraction of paused transcripts is shown. The line generated using robust linear regression (RLM function in Python), shows very weak correlation of RNA Pol II pausing with stable DNA secondary structures.

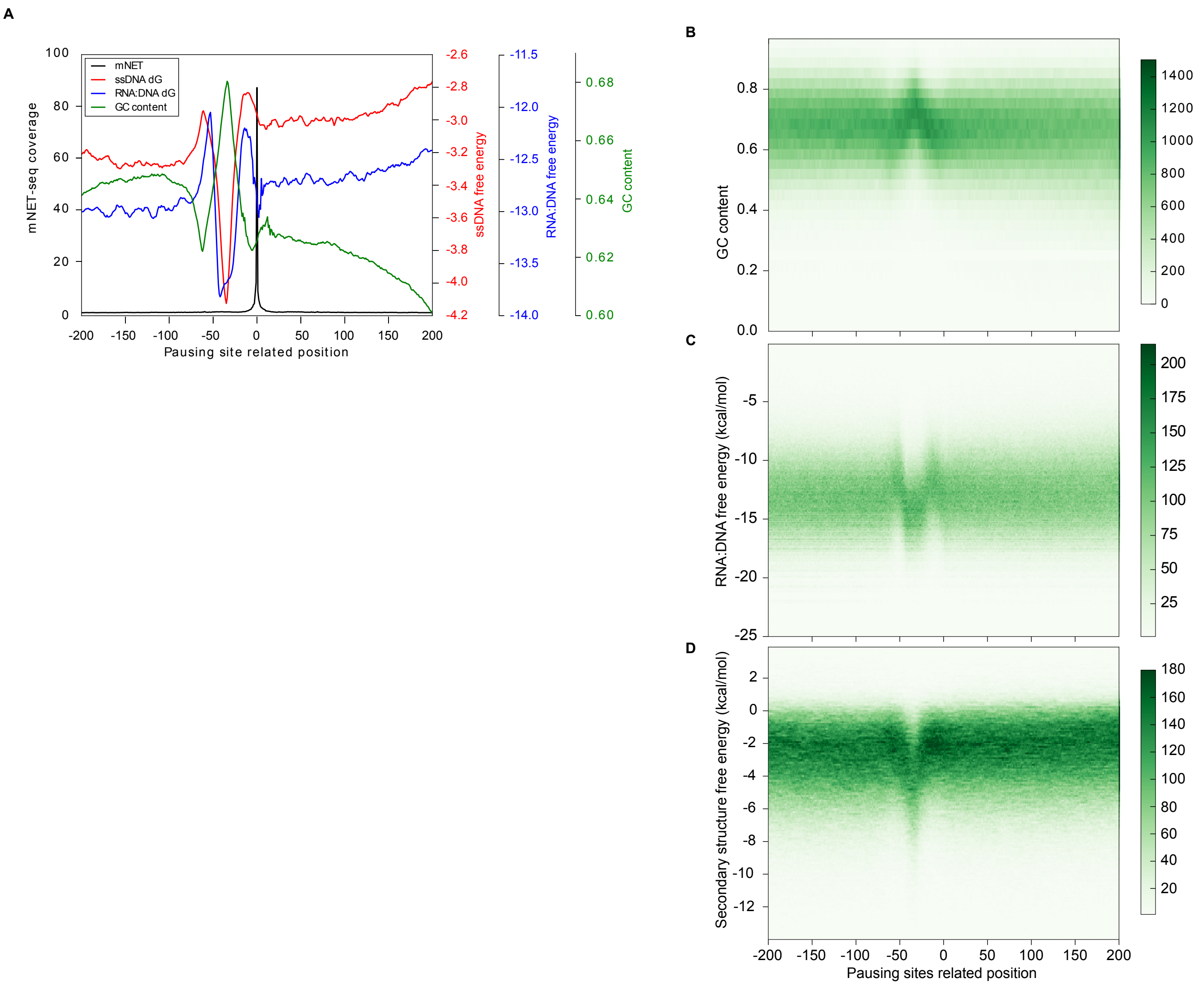

Figure S13. Genomic features at Pol II pausing sites. A. Average plots of non-template strand secondary structure free energy (red), RNA:DNA hybrid free energy (blue), GC content (green), and mNET-seq coverage (black) are shown for the genes with pausing sites within gene bodies (n = 7972). Frequency plots presenting individual genes of **A** are shown: GC content (**B**) RNA:DNA hybrid free energy (**C**), and free energy of stem-loop secondary structure (**D**). The "0" pausing site-related position on the x-axis is the nucleotide where the highest mNET-seq read spike is in each pausing site.

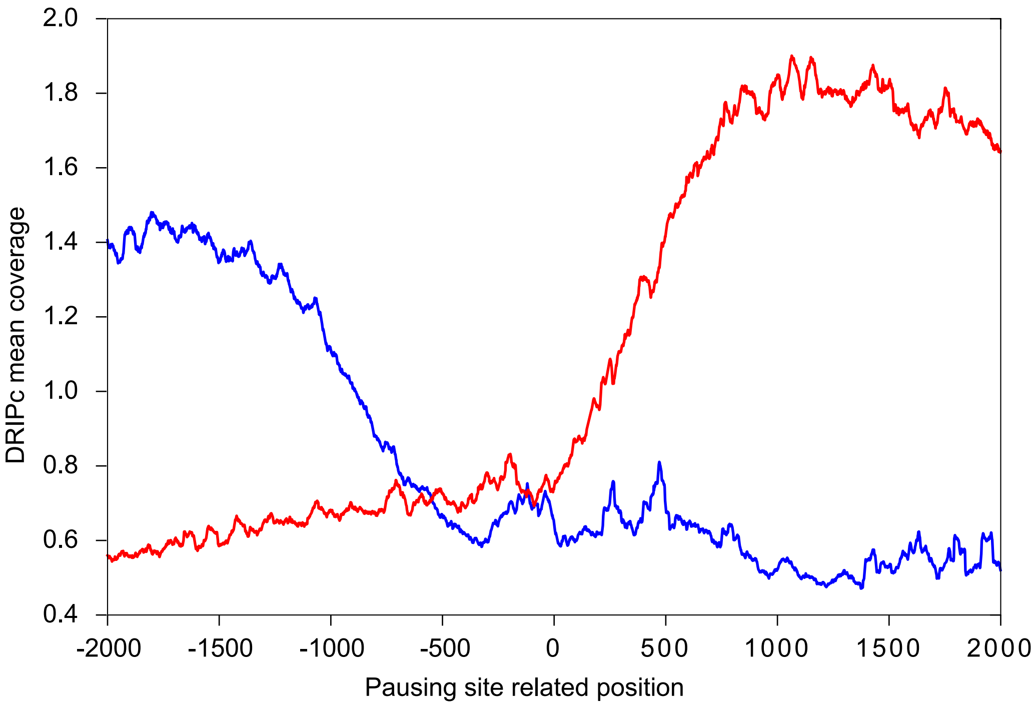

Figure S14. R-loops at pausing sites. R-loops, as measured by DRIPc-seq (Sanz et al. 2016) and the position of pausing sites defined by HeLa-S3 mNET-seq data (Nojima et al. 2015), are shown at all pausing sites ± 200 nt for non-template (red line) and template (blue line) strands (n=13931). Note: no DRIP-seq data is available in HeLa cells. Because of a high correlation of pausing patterns among cell lines, it is reasonable to use pausing sites obtained in HeLa cells and R-loop signals from Ntera2 cells. The "0" pausing site-related position on the x-axis is the nucleotide where the highest mNET-seq read spike is in each pausing site.

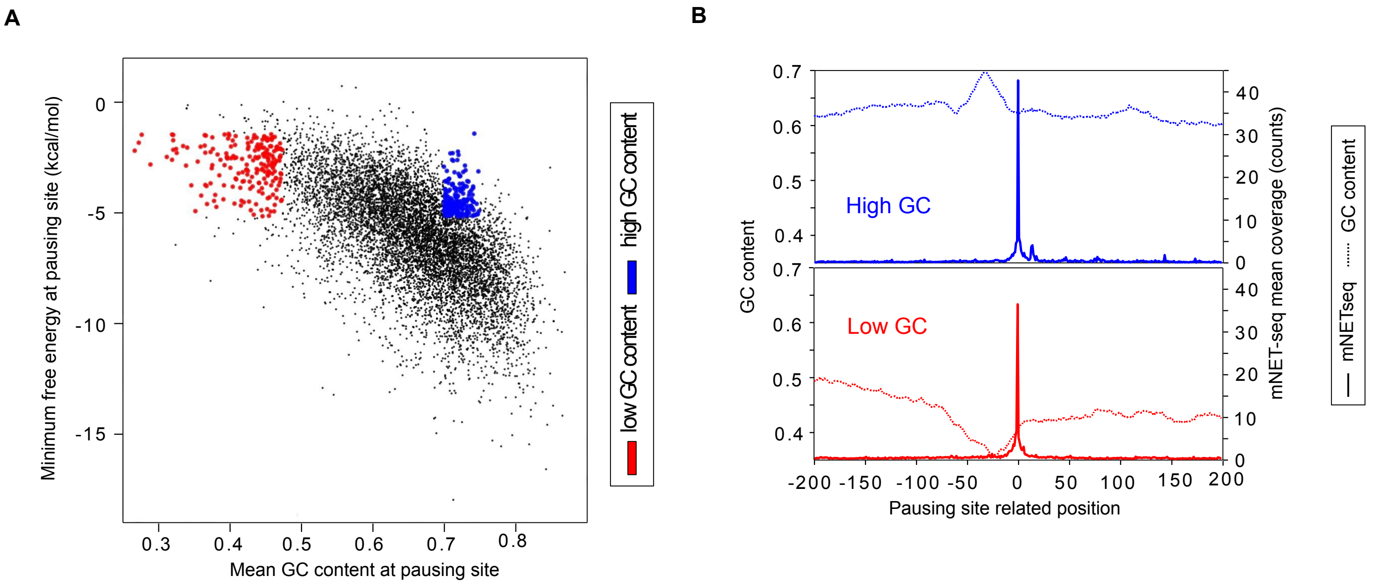

Figure S15. GC content and Pol II pausing sites. A. Scatter plot shows minimum free energy vs. mean GC content at a region -50 nt to -10 nt from the pausing site. Pausing sites with a high (0.70 to 0.75, n=200, blue dots) or low (<0.48, n=200, red dots) GC content, but with matching free energies (>-5 kcal/mol) are identified. **B.** Average profiles of GC content (dotted line) and mNET-seq coverage (solid line) are shown for the high (blue) and low GC (red) groups identified in **A**. Both groups showed a similar pausing pattern.

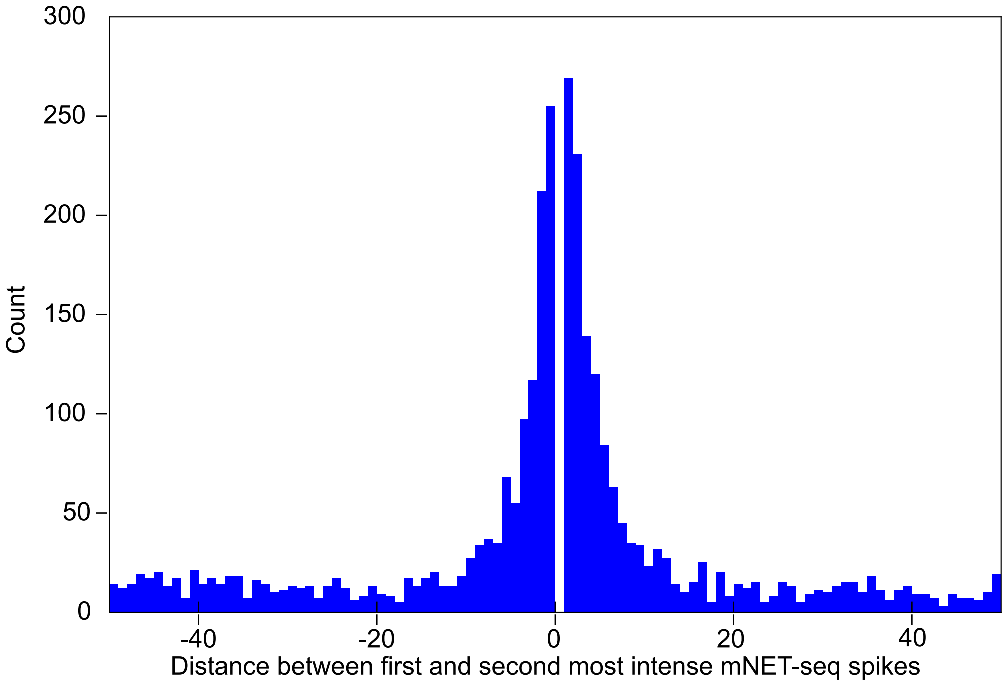

Figure S16. Trace of backtracked polymerase. Histogram presents the distance of the second most intense mNET-seq spike related to the most intense mNET-seq spike at pausing sites within genes (n = 5263). Pausing sites are defined as the most intense mNET-seq spike.

Supplemental Methods and References

Supplemental Methods:

Two-Dimensional Frequency Plots:
Two-dimensional frequency plots were generated in Python 3.5 with pandas (v. 0.18.1), numpy (v. 1.11.1) and matplotlib (v. 1.5.3).

Stability of RNA:DNA hybrids:
Stability of RNA:DNA hybrids is calculated with a subversion of ViennaRNA dedicated to calculate RNA:DNA hybrids free energy (Lorenz et al. 2012).

Supplemental References:

Lorenz R, Hofacker IL, Bernhart SH. 2012. Folding RNA/DNA hybrid duplexes. *Bioinformatics* 28(19):2530-2531.

Nojima T, Gomes T, Grosso AR, Kimura H, Dye MJ, Dhir S, Carmo-Fonseca M, Proudfoot NJ. 2015. Mammalian NET-Seq Reveals Genome-wide Nascent Transcription Coupled to RNA Processing. *Cell* 161(3): 526-540.

Sanz LA, Hartono SR, Lim YW, Steyaert S, Rajpurkar A, Ginno PA, Xu X, Chedin F. 2016. Prevalent, Dynamic, and Conserved R-Loop Structures Associate with Specific Epigenomic Signatures in Mammals. *Mol Cell* **63**(1): 167-178.
